# Intraindividual double burden of overweight and micronutrient deficiencies or anemia among preschool children

**DOI:** 10.1093/ajcn/nqaa101

**Published:** 2020-08-04

**Authors:** Reina Engle-Stone, Junjie Guo, Sanober Ismaily, O Yaw Addo, Tahmeed Ahmed, Brietta Oaks, Parminder S Suchdev, Rafael Flores-Ayala, Anne M Williams

**Affiliations:** Department of Nutrition, University of California, Davis, CA, USA; Hubert Department of Global Health, Emory University, Atlanta, GA, USA; Hubert Department of Global Health, Emory University, Atlanta, GA, USA; Hubert Department of Global Health, Emory University, Atlanta, GA, USA; McKing Consulting Corporation, Atlanta, GA, USA; Nutrition & Clinical Services Division, icddr,b, Dhaka, Bangladesh; Department of Nutrition and Food Sciences, University of Rhode Island, Kingston, RI, USA; Hubert Department of Global Health, Emory University, Atlanta, GA, USA; Emory Global Health Institute and Department of Pediatrics, Emory University, Atlanta, GA, USA; Division of Nutrition, Physical Activity and Obesity, US CDC, Atlanta, GA, USA; Division of Nutrition, Physical Activity and Obesity, US CDC, Atlanta, GA, USA; Hubert Department of Global Health, Emory University, Atlanta, GA, USA; McKing Consulting Corporation, Atlanta, GA, USA

**Keywords:** double burden of malnutrition, preschool children, anemia, overweight/obesity, micronutrients

## Abstract

**Background:**

Child overweight prevalence is increasing globally, but micronutrient deficiencies persist.

**Objectives:**

We aimed to *1*) describe the prevalence and distribution of intraindividual double burden of malnutrition (DBM), defined as coexistence of overweight or obesity (OWOB) and either micronutrient deficiencies or anemia, among preschool children; *2*) assess the independence of DBM components, e.g., whether the prevalence of DBM is greater than what would be expected by chance; and *3*) identify predictors of intraindividual DBM, to guide intervention targeting.

**Methods:**

We analyzed data from 24 population-based surveys from the Biomarkers Reflecting Inflammation and Nutritional Determinants of Anemia project (separately by survey; *n* = 226 to *n* = 7166). We defined intraindividual DBM as coexisting OWOB and ≥1 micronutrient deficiency [e.g., Micronutrient Deficiency Index (MDI) > 0; DBM-MDI] or anemia (DBM-Anemia). We assessed independence of DBM components with the Rao–Scott chi-square test and examined predictors of DBM and its components with logistic regression.

**Results:**

DBM prevalence ranged from 0% to 9.7% (median: 2.5%, DBM-MDI; 1.4%, DBM-Anemia), reflecting a lower prevalence of OWOB (range: 0%–19.5%) than of micronutrient deficiencies and anemia, which exceeded 20% in most surveys. OWOB was generally not significantly associated with micronutrient deficiencies or anemia. In more than half of surveys, children 6–23 mo of age, compared with ≥24 mo, had greater adjusted odds of DBM-Anemia, anemia, and micronutrient deficiencies. Child sex and household socioeconomic status, urban location, and caregiver education did not consistently predict DBM or its components.

**Conclusions:**

Intraindividual DBM among preschool children was low but might increase as child OWOB increases. The analysis does not support the hypothesis that DBM components cluster within individuals, suggesting that population-level DBM may be addressed by programs to reduce DBM components without targeting individuals with DBM.

## Introduction

The prevalence and number of children affected by overweight and obesity have risen dramatically over the past several decades (1,2). An estimated 41 million preschool children were overweight in 2014 (3); these children are at increased risk of overweight later in life (4). At the same time, undernutrition remains a persistent problem, especially in low- and middle-income countries where food insecurity and low dietary diversity are common (5,6). An estimated 29% of preschool children have vitamin A deficiency (7) and up to ∼18% may have anemia amenable to iron provision (8); 17% of the population are at risk of inadequate zinc intake (9). Many low- and middle-income countries are undergoing the “nutrition transition,” which is characterized by a shift from food shortage and dietary insufficiency to caloric surplus and increased risk of noncommunicable disease (NCD) (10). “Double-duty actions,” e.g., intervention programs that are expected to positively affect aspects of both under- and over-nutrition, have been suggested to address multiple forms of malnutrition (11,12), although experience implementing programs with these explicit joint objectives is limited.

Efforts to monitor the prevalence of the double burden of malnutrition (DBM) are necessary to inform the development and targeting of interventions to address all forms of malnutrition. The WHO defines the DBM as “the coexistence of undernutrition along with overweight, obesity or diet-related NCDs, within individuals, households and populations, and across the life-course” and notes that undernutrition may refer to wasting, stunting, and micronutrient deficiencies (13). Previous research has examined the DBM using available anthropometric data alone [e.g., stunting and overweight (14)] or in combination with hemoglobin concentration (15,16) or, less commonly, biomarkers of micronutrient deficiency (17). There is no consensus on operational definitions of the DBM. Choice of indicator (e.g., selecting stunting, wasting, or iron deficiency as the indicator of undernutrition, or BMI compared with indicators of diet-related NCD, such as blood pressure) as well as the level of assessment (individual, household, or population) is likely to influence the prevalence and distribution of the DBM, and hence the policy conclusions that will be drawn from these estimates. Whereas anthropometric indicators and hemoglobin concentration are commonly measured, few large, population-based surveys collect data on individual micronutrient status. Thus, the prevalence of DBM with respect to micronutrient deficiencies, particularly among young children, is less well documented.

Several factors may give rise to coexistent overweight or obesity (OWOB) and undernutrition. At the population level, presence of both conditions may reflect divergent diet and lifestyle patterns in population subgroups—for example, increased consumption of energy-dense foods and sedentary behavior in urban areas, and food insecurity and undernutrition in agriculture-dependent rural areas. At the individual level, dietary patterns that are energy-dense and low in micronutrients, e.g., frequent consumption of ultra-processed snack foods or sugar-sweetened beverages, may contribute to both overweight and micronutrient deficiencies or anemia (18). Obesity may also affect micronutrient metabolism in ways that influence risk of deficiency, such as reduced iron absorption reflecting inflammation-induced increases in circulating hepcidin (19). An alternative explanation for the overlap is simply mathematical: if childhood overweight and undernutrition (e.g., micronutrient deficiencies) are independent conditions, some overlap would still be expected due to chance, as observed for stunted child–overweight mother pairs (20). Characterizing the intraindividual DBM among children is the critical first step to assess the utility of specifically targeting such individuals with intervention programs and to inform the development of appropriate interventions.

To address these gaps, the objectives of this study were to *1*) describe the prevalence and distribution of intraindividual DBM, defined as coexistence of OWOB and either micronutrient deficiencies or anemia, among preschool children in diverse settings; *2*) assess the independence of DBM components, e.g., whether the prevalence of DBM is greater than what would be expected by chance; and *3*) identify predictors of intraindividual DBM, to guide intervention targeting.

## Methods

### Data source and inclusion criteria

We used data from the Biomarkers Reflecting Inflammation and Nutritional Determinants of Anemia (BRINDA) project (www.brinda-nutrition.org). The BRINDA data set was compiled using data on preschool children from 24 population-based surveys (21 national and 3 regional) from 21 countries. Inclusion criteria for surveys and methods of harmonization are described elsewhere (21). Briefly, observations were eligible for inclusion in the current analysis if they had available data for BMI-for-age *z* score (BAZ) and weight-for-height *z* score (WHZ), hemoglobin, a marker of inflammation [C-reactive protein (CRP) or α-1-acid glycoprotein (AGP)], and ≥1 micronutrient status indicator {ferritin, retinol-binding protein, retinol, zinc, vitamin B-12, folate, red blood cell folate (RBCF), or 25-hydroxyvitamin D [25(OH)D]}. Due to substantial heterogeneity in survey populations, all analyses were conducted separately for each survey. However, we present selected results with surveys grouped into WHO geographic regions: Southeast Asia/Western Pacific, Africa, Americas, and Europe/Eastern Mediterranean.

Child weight and height or length were measured in all surveys, and anthropometric *z* scores were recalculated for the BRINDA database using the WHO growth standards (21). We excluded observations with implausible values for BAZ or WHZ (<−5 SD or >5 SD). Hemoglobin was measured in capillary blood (except in Azerbaijan, Cameroon, Cote d'Ivoire, Malawi, and the USA, where venous blood was collected); all but 4 surveys used the Hemocue instrument. Hemoglobin concentration was adjusted for altitude where data allowed (22). **Supplemental Tables 1** and **2** present original survey references and information on the micronutrient status indicators measured and the corresponding laboratory methods used in each survey.

### Outcome definitions

The primary outcomes were the prevalence of intraindividual DBM (using varying definitions, as described below) and each of the DBM components (OWOB, micronutrient deficiencies, and anemia).

We defined intraindividual DBM in several ways. First, to summarize information on the total burden of micronutrient deficiencies at the individual level, we created a composite Micronutrient Deficiency Index (MDI) score and examined overlap between OWOB (BAZ > 2 SD) and the dichotomized MDI score. In addition, we examined the overlap between OWOB and single micronutrient deficiencies (OWOB + iron deficiency, OWOB + vitamin A deficiency, etc.). Lastly, although anemia is not an indicator of micronutrient status (or nutritional status) per se, it is frequently used as a proxy measure, including in studies of DBM, and is often included in national surveys, such as Demographic and Health Surveys. Thus, for comparison, we also defined the DBM as OWOB and anemia (DBM-Anemia).

We defined OWOB as BAZ > 2 SD, and “at risk of overweight” was defined as BAZ > 1 SD and ≤2 SD (23). WHZ is recommended for use among children <24 mo of age (23), but using WHZ to define OWOB yielded similar results, and thus we applied BAZ to all age groups for consistency.

Anemia was defined as whole blood hemoglobin concentration < 110 g/L. The following cutoffs were used to indicate deficiency for each of the micronutrients assessed: *1*) iron: inflammation-adjusted serum or plasma ferritin < 12 µg/L (24); *2*) vitamin A: inflammation-adjusted serum or plasma retinol-binding protein or retinol < 0.70 µmol/L (25); *3*) vitamin D: serum or plasma 25(OH)D < 30 nmol/L (referred to as risk of deficiency by the US Institute of Medicine) (26); *4*) folate: RBCF < 226.5 nmol/L (if measured using the microbiologic assay) or serum or plasma folate < 6.8 nmol/L (27); *5*) vitamin B-12: serum or plasma vitamin B-12 < 150 pmol/L (28); and *6*) zinc: serum or plasma zinc concentration < 650 µg/L for nonfasting morning samples and <570 µg/L for nonfasting afternoon samples (29). Serum or plasma zinc values were adjusted for inflammation in 6 surveys (Afghanistan, Azerbaijan, Cambodia, Cameroon, Ecuador, and Malawi) based on significant correlations between zinc and either CRP or AGP, and clear patterns of increasing prevalence of low zinc at higher deciles of CRP or AGP (30).

The MDI was defined as the number of micronutrients for which biomarker values indicated low status (i.e., individuals could receive a score between 0, indicating no evidence of micronutrient deficiency, and 6, the maximum number of micronutrients assessed in an individual survey). To estimate the prevalence of intraindividual DBM, we dichotomized the ordinal MDI score into a binary outcome representing no micronutrient deficiencies (MDI = 0) or ≥1 micronutrient deficiencies (MDI ≥ 1). In addition, to provide descriptive information on the frequency of occurrence of 1 compared with multiple micronutrient deficiencies, as a post hoc analysis we expressed the MDI as a 3-category variable representing no micronutrient deficiencies (MDI = 0), 1 micronutrient deficiency (MDI = 1), or multiple micronutrient deficiencies (MDI > 1).

To examine predictors of the DBM, we defined the following individual and household characteristics: child age (≥24.0 mo compared with 6–23.9 mo), child sex, urban location (as defined by each survey representative, compared with rural location), low caregiver education (defined as no formal education or primary-level education, compared with secondary or higher education; educational level of the head of household was substituted where caregiver education was not available), and socioeconomic status (SES), defined as an ordinal 3-category variable (21).

### Statistical analyses

Analyses were conducted in SAS version 9.4 (SAS Institute) using survey procedures and weights to generate appropriate variance estimates for complex survey designs. Mongolia did not use a complex sampling design, so variance estimates were generated using a binomial distribution. All analyses were conducted separately for each survey by 2 independent analysts; discrepancies were resolved through discussion and consensus. We calculated the expected prevalence of DBM as the product of the DBM components: specifically, prevalence of OWOB and *1*) each individual micronutrient deficiency, *2*) any micronutrient deficiency (MDI > 0), or *3*) anemia. We tested independence of OWOB and each measure of undernutrition using the Rao–Scott chi-square test to account for the cluster survey design. Predictors of the DBM in each survey were assessed using logistic regression (PROC SURVEYLOGISTIC in SAS), excluding surveys with *n* < 10 observations with DBM (DBM-MDI: Bangladesh 2010, Bangladesh 2012, Cambodia, Georgia, Laos, Papua New Guinea, and Vietnam; DBM-Anemia: Bangladesh 2012, Cambodia, Laos, Mongolia, USA, and Vietnam). According to our analysis plan, we first examined bivariate relations between DBM-MDI and DBM-Anemia and each potential predictor variable and then conducted adjusted logistic regression analyses using a model with all the conceptual predictors available in a given survey data set: age group, child sex, urban compared with rural location, SES, and maternal education. If there were no cases of DBM in 1 of the levels, the variable was collapsed into 2 categories (in the case of SES) or excluded from the model. In addition, child age was excluded from models for Bangladesh 2010 and the Philippines because those surveys only recruited children <24 mo of age. After observing the results of the independence testing, we included a post hoc analysis to assist in interpreting the predictors of DBM; namely, we used adjusted logistic regression models to assess predictors of each of the DBM components: micronutrient deficiency (MDI > 0), anemia, and OWOB.

### Ethical approval and role of the funding source

The BRINDA study was reviewed by the institutional review board of the NIH (protocol #11417) and deemed non–human subjects research.

## Results

### Participant characteristics and prevalence of DBM and DBM components

Data from 34,654 children in 24 surveys from 21 countries were included in the analysis (**Table 1**). The majority of surveys included children 6–59 mo or 12–59 mo of age, but several recruited younger children, notably in Bangladesh 2010 (6–11 mo) and the Philippines (6–24 mo).

**TABLE 1:** Age, sex, and household characteristics of preschool children by survey: Biomarkers Reflecting Inflammation and Nutritional Determinants of Anemia project^1^

Geographic grouping	Country, survey year	*n*	Age,^2^ mo	Males	Urban residence	High SES	High education^3^
Southeast Asia/Western Pacific	Bangladesh, 2010	1473	8.3 (6, 11)	49.2 (46.9, 51.4)	—	—	—
	Bangladesh, 2012	429	36.2 (6, 59)	43.6 (33.7, 53.5)	24.4 (16.7, 32.2)	17.7 (9.6, 25.9)	46.0 (35.4, 56.6)
	Cambodia, 2014	406	35.8 (6.1, 59.9)	56.3 (50.8, 61.8)	11.9 (8.3, 15.5)	14.1 (8.2, 20.0)	25.8 (20.7, 30.8)
	Laos, 2006	478	33.1 (6, 59)	49.4 (44.8, 54.0)	13.9 (5.4, 22.5)	11.7 (5.1, 18.2)	15.5 (7.8, 23.1)
	Mongolia, 2006	226	20.3 (6.4, 35.8)	51.3 (44.8, 57.8)	46.9 (40.4, 53.4)	—	80.5 (75.3, 85.8)
	PNG, 2005	844	31.4 (6.0, 59.8)	54.5 (51.3, 57.7)	19.4 (10.0, 28.8)	26.3 (16.6, 35.9)	—
	Philippines, 2011	1762	15.0 (6.0, 23.9)	50.0 (47.1, 52.9)	9.1 (8.4, 9.7)	0.1 (0, 0.3)	66.3 (61.2, 71.3)
	Vietnam, 2010	373	37.3 (10.4, 59.7)	53.6 (48.9, 58.3)	46.9 (41.3, 52.5)	—	—
Africa	Cote d'Ivoire, 2007	708	31.6 (6, 59)	53.9 (49.7, 58.1)	50.0 (45.1, 54.9)	19.6 (15.0, 24.2)	12.2 (9.5, 15.0)
	Cameroon, 2009	742	31.0 (12, 59.9)	50.6 (47.1, 54.2)	58.5 (48.5, 68.4)	15.1 (12.2, 18.0)	31.8 (27.7, 35.9)
	Kenya, 2007	862	19.9 (6.0, 35.9)	52.7 (49.5, 55.8)	0	20.4 (16.8, 24.1)	12.5 (10.0, 15.0)
	Kenya, 2010	838	21.4 (6, 35)	50.2 (46.7, 53.7)	0	17.5 (13.8, 21.1)	15.7 (12.3, 19.1)
	Liberia, 2011	1429	19.9 (6, 35.9)	50.3 (47.4, 53.1)	37.5 (34.0, 41.0)	22.7 (19.4, 26.0)	—
	Malawi, 2016	1070	32.5 (6, 59)	49.2 (46.1, 52.2)	10.4 (0, 21.6)	9.9 (4.8, 15.0)	20.5 (11.6, 29.5)
Americas	Colombia, 2010	3808	37.7 (12, 59)	52.1 (50.0, 54.1)	69.9 (68.6, 71.2)	8.6 (7.2, 10.0)	42.6 (39.7, 45.6)
	Ecuador, 2012	1931	30.8 (6, 59)	49.2 (45.1, 53.3)	65.3 (53.2, 77.3)	13.1 (9.3, 16.8)	67.3 (62.9, 71.6)
	Mexico, 2006	1589	41.6 (12.8, 59.9)	51.3 (47.5, 55.1)	53.1 (48.8, 57.4)	6.9 (4.5, 9.3)	37.7 (33.7, 41.7)
	Mexico, 2012	2178	39.2 (12, 59.9)	49.3 (46.6, 52.1)	66.3 (64.1, 68.5)	11.4 (9.4, 13.3)	—
	Nicaragua, 2005	1419	33.4 (6.1, 59.9)	49.6 (46.3, 52.9)	56.4 (44.1, 68.7)	—	37.2 (30.0, 44.3)
	USA, 2006	1242	37.0 (12, 59)	52.1 (49.4, 54.8)	—	20.6 (16.4, 24.8)	93.5 (91.7, 95.3)
Europe/Eastern Mediterranean	Afghanistan, 2013	585	29.2 (6, 58)	56.5 (51.0, 61.9)	—	69.2 (61.1, 77.4)	—
	Azerbaijan, 2013	1015	35.8 (6, 59)	54.8 (50.5, 59.1)	45.2 (38.0, 52.4)	24.1 (19.4, 28.8)	—
	Georgia, 2009	2081	36.5 (12.0, 59.9)	53.1 (50.4, 55.8)	47.0 (40.2, 53.8)	—	—
	Pakistan, 2011	7166	27.5 (6, 59)	51.9 (50.4, 53.3)	30.0 (27.0, 32.9)	16.7 (14.9, 18.4)	19.3 (17.7, 21.0)

1 Values are percentages (95% CIs) unless otherwise indicated; estimates account for survey design. Mongolia did not use a complex sampling design, so the 95% CI was generated using a binomial distribution. —, variable (or category) was unavailable in that survey. PNG, Papua New Guinea; SES, socioeconomic status.

2 Mean (range).

3 Low education defined as no education or primary education of the child's caregiver. Education level of the head of household was used in place of caregiver education in surveys in which caregiver education was not measured (Colombia, Mexico 2006, and the USA).

**FIGURE 1:**
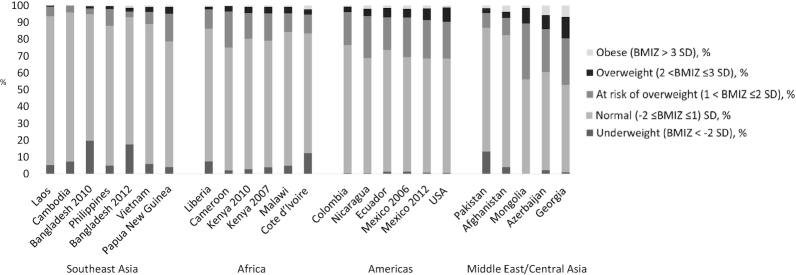
Distribution of BAZ among preschool children, by survey, Biomarkers Reflecting Inflammation and Nutritional Determinants of Anemia project. Shaded bars indicate the proportion of children in each survey categorized as underweight (BAZ < −2 SD), normal weight (−2 ≤ BAZ ≤ 1), at risk of overweight (1 < BAZ ≤ 2 SD), overweight (2 < BAZ ≤ 3 SD), and obese (BAZ > 3 SD). BAZ, BMI-for-age *z* score.

The prevalence of OWOB (BAZ > 2 SD) ranged from 0% in Cambodia to 19.5% in Georgia (median = 4.6%), and exceeded 5% in 10 surveys (**Figure 1**). The prevalence of BAZ > 1 SD (children considered OWOB or “at risk of overweight”) ranged from 4% in Cambodia (below the expected 15.9% in the reference population) to >40% in Mongolia and Georgia. Although there was substantial variation by survey, BAZs tended to be greater in surveys conducted in the Americas and Europe/Eastern Mediterranean than in Southeast Asia/Western Pacific (Figure 1). Although we focused on micronutrient deficiency as the indicator of undernutrition, we noted that the prevalence of wasting was >10% in Bangladesh (both surveys), Cote d'Ivoire, and Pakistan (Figure 1 reports prevalence of BAZ < −2 SD; results for WHZ < −2 SD were similar; data not shown).

Micronutrient deficiencies were more common than OWOB, although the prevalence varied by survey and nutrient (**Table 2**). Most surveys measured iron (23 of 24 surveys) and vitamin A status (19 surveys), but half or fewer measured zinc (12 surveys) or other micronutrients (8, folate; 6, vitamin D; 4, vitamin B-12). The prevalence of children with 0 micronutrient deficiencies identified ranged from only 7% in Mongolia (in which 5 micronutrients were measured) to almost 100% in Georgia (in which only iron status was measured). The proportion of children with a single micronutrient deficiency (e.g., MDI = 1) was generally greater than the proportion of children with multiple deficiencies, except in Afghanistan, Mongolia, and Pakistan (all of which measured ≥4 micronutrients). Prevalence of anemia ranged from 1.9% in the USA to 83.4% in Bangladesh 2010 (median = 35.8%), with the highest values observed in Africa and Southeast Asia. At the survey level, anemia prevalence was not significantly associated with prevalence of MDI > 0 (*r*_s _= 0.19, *P* = 0.37, *n* = 24).

**TABLE 2:** Prevalence of micronutrient deficiencies and anemia among preschool children by survey: Biomarkers Reflecting Inflammation and Nutritional Determinants of Anemia project^1^

		MDI, %			Prevalence of individual micronutrient deficiency, %						
Country, survey year	# MN	0	1	>1	Iron	Vitamin A	Zinc	Vitamin B-12	Folate	Vitamin D	Anemia
Cambodia, 2014	6	39.0 (30.5, 47.6)	48.9 (41.4, 56.4)	12.0 (8.0, 16.1)	5.4 (2.9, 7.9)	5.6 (3.3, 7.9)	62.8 (53.8, 71.7)	2.1 (0.6, 3.6)	6.0 (3.2, 8.8)	4.7 (1.3, 8.0)	53.3 (47.4, 59.2)
Cameroon, 2009	5	23.8 (20.3, 27.4)	46.7 (43.3, 50.1)	29.5 (25.9, 33.0)	34.9 (30.7, 39.1)	9.9 (7.4, 12.4)	58.5 (53.6, 63.3)	15.4 (10.6, 20.1)	8.5 (5.0, 11.9)	—	54.9 (49.9, 59.9)
Mongolia, 2006	5	7.1 (3.7, 10.4)	32.7 (26.7, 38.9)	60.2 (53.8, 66.6)	43.8 (37.3, 50.3)	26.8 (20.6, 33.0)	78.8 (73.4, 84.1)	—	16.3 (11.3, 21.2)	71.7 (62.5, 80.9)	24.3 (18.7, 29.9)
Vietnam, 2010	5	26.5 (21.8, 31.3)	50.7 (44.8, 56.5)	22.8 (18.1, 27.5)	18.5 (14.6, 22.4)	5.6 (3.1, 8.1)	56.5 (51.0, 62.0)	—	4.1 (1.0, 7.1)	20.8 (15.9, 25.8)	7.5 (4.4, 10.6)
Afghanistan, 2013	4	25.3 (20.3, 30.4)	35.9 (30.7, 41.0)	38.8 (34.2, 43.4)	23.0 (18.3, 27.7)	38.8 (33.3, 44.3)	23.1 (17.8, 28.3)	—	—	43.4 (37.2, 49.6)	39.8 (33.1, 46.5)
Colombia, 2010	4	41.3 (39.1, 43.4)	45.0 (42.8, 47.1)	13.8 (12.4, 15.1)	13.6 (12.1, 15.0)	17.6 (15.9, 19.4)	44.8 (42.6, 47.1)	—	—	—	13.5 (12.0, 15.1)
Ecuador, 2012	4	58.9 (55.6, 62.2)	32.2 (29.9, 34.5)	8.9 (6.6, 11.2)	12.7 (10.2, 15.1)	—	26.3 (23.7, 28.9)	—	0.9 (0.2, 1.6)	—	22.3 (18.7, 26.0)
Mexico, 2006	4	51.6 (47.6, 55.6)	37.8 (34.1, 41.5)	10.6 (8.4, 12.9)	35.1 (31.5, 38.7)	—	28.2 (24.1, 32.3)	2.6 (1.1, 4.0)	4.2 (2.4, 6.0)	—	20.7 (17.5, 23.8)
Mexico, 2012	4	75.9 (73.3, 78.4)	21.9 (19.5, 24.2)	2.3 (1.4, 3.1)	18.5 (15.7, 21.4)	7.3 (5.6, 9.0)	—	0.3 (0.1, 0.4)	0.3 (0.0, 0.7)	—	18.5 (15.6, 21.4)
Pakistan, 2011	4	10.8 (9.8, 11.8)	34.3 (32.9, 35.8)	54.9 (53.1, 56.7)	51.6 (50.0, 53.2)	51.7 (49.5, 54.0)	49.6 (47.6, 51.7)	—	—	18.2 (16.7, 19.8)	63.2 (61.8, 64.5)
USA, 2006	4	85.7 (82.5, 88.9)	13.8 (10.6, 16.9)	0.6 (0.1, 1.0)	12.9 (9.8, 16.1)	—	—	—	2.5 (1.9, 3.1)	0.8 (0.4, 1.3)	1.9 (0.8, 3.0)
Azerbaijan, 2013	3	67.0 (63.1, 70.8)	28.8 (25.2, 32.4)	4.2 (2.7, 5.6)	22.0 (18.3, 25.7)	6.6 (4.1, 9.1)	9.2 (7.2, 11.3)	—	—	—	28.6 (24.5, 32.7)
Bangladesh, 2012	3	54.8 (46.4, 63.2)	36.8 (27.4, 46.2)	8.3 (2.9, 13.8)	11.6 (6.4, 16.8)	10.0 (4.2, 15.7)	47.3 (36.1, 58.5)	—	—	—	31.8 (23.0, 40.7)
Malawi, 2016	3	35.8 (30.9, 40.7)	51.2 (46.8, 55.5)	13.0 (9.8, 16.2)	21.9 (16.4, 27.4)	7.9 (5.3, 10.4)	60.8 (54.8, 66.8)	—	—	—	30.8 (27.0, 34.5)
Bangladesh, 2010	2	79.0 (76.2, 81.7)	20.0 (17.4, 22.6)	1.1 (0.4, 1.7)	16.0 (13.4, 18.5)	6.2 (4.2, 8.2)	—	—	—	—	83.4 (81.0, 85.8)
Cote d'Ivoire, 2007	2	59.0 (54.8, 63.2)	40.1 (36.0, 44.2)	0.9 (0.2, 1.6)	38.9 (35.0, 42.9)	3.0 (1.7, 4.4)	—	—	—	—	71.9 (66.8, 76.9)
Kenya, 2007	2	25.3 (21.8, 28.8)	70.2 (66.6, 73.7)	4.5 (3.2, 5.9)	73.0 (69.3, 76.6)	6.3 (4.2, 8.3)	—	—	—	—	67.1 (63.1, 71.0)
Kenya, 2010	2	42.5 (38.9, 46.1)	53.1 (49.6, 56.6)	4.4 (3.1, 5.8)	53.7 (50.0, 57.4)	8.2 (6.6, 9.8)	—	—	—	—	71.5 (67.9, 75.1)
Liberia, 2011	2	46.8 (42.7, 50.8)	50.1 (46.4, 53.7)	3.2 (2.1, 4.3)	51.1 (47.1, 55.0)	5.3 (3.9, 6.8)	—	—	—	—	59.5 (55.6, 63.4)
Nicaragua, 2005	2	77.1 (70.3, 83.9)	22.5 (15.8, 29.2)	0.4 (0.1, 0.8)	44.9 (38.2, 51.6)	0.8 (0.3, 1.3)	—	—	—	—	20.3 (15.7, 24.8)
Philippines, 2011	2	64.6 (61.1, 68.2)	34.8 (31.3, 38.3)	0.5 (0.1, 0.9)	34.9 (31.4, 38.4)	0.9 (0.4, 1.5)	—	—	—	—	41.9 (37.8, 45.9)
Georgia, 2009	1	99.8 (99.6, 100)	0.2 (0.0, 0.4)	0.0	0.2 (0.0, 0.4)	—	—	—	—	—	23.1 (19.8, 26.4)
Laos, 2006	1	74.4 (69.6, 79.1)	25.6 (20.9, 30.4)	0.0	25.6 (20.9, 30.4)	—	—	—	—	—	40.5 (31.0, 50.0)
PNG, 2005	1	88.4 (86.0, 90.8)	11.6 (9.2, 14.0)	0.0	—	11.6 (9.2, 14.0)	—	—	—	—	48.1 (42.4, 53.9)

1 Values represent *n* or % (95% CI), unless otherwise indicated. Surveys in descending order of # MN and ascending order of MDI = 0. Estimates take into account survey design variables (cluster, strata, weight). Mongolia did not use a complex sampling design, so the 95% CI was generated using a binomial distribution. Cutoffs to define deficiency: iron, inflammation-adjusted ferritin < 12 µg/L; vitamin A, inflammation-adjusted retinol-binding protein or retinol < 0.7 μmol/L; zinc, serum or plasma zinc < 650 µg/L for nonfasting children in the morning or <570 µg/L for nonfasting children in the afternoon/evening (adjusted for inflammation in Afghanistan, Azerbaijan, Cambodia, Cameroon, and Ecuador); folate, RBC folate < 226.5 nmol/L (if measured using the microbiologic assay) or serum or plasma folate < 6.8 nmol/L; vitamin B-12, serum or plasma vitamin B-12 < 150 pmol/L; vitamin D, 25-hydroxyvitamin D < 30 nmol/L. MDI was defined as the number of micronutrient biomarkers with values below the threshold used to define risk of deficiency. MDI, Micronutrient Deficiency Index; PNG, Papua New Guinea; # MN, number of micronutrients measured.

The prevalence of intraindividual DBM ranged from 0% (Cambodia) to 9.7% (Mongolia) for DBM-MDI (median = 2.6%) and from 0% (Cambodia) to 5.0% (Georgia) for DBM-Anemia (median = 1.4%) (**Table 3**).

**TABLE 3:** Prevalence estimates of concomitant overweight/obesity and micronutrient deficiencies or anemia among preschool children by survey: Biomarkers Reflecting Inflammation and Nutritional Determinants of Anemia project^1^

		Anemia		MDI > 0		Iron		Vitamin A		Zinc		Folate		Vitamin D		Vitamin B-12	
Geographic grouping	Country, survey year	Obs.	Exp.	Obs.	Exp.	Obs.	Exp.	Obs.	Exp.	Obs.	Exp.	Obs.	Exp.	Obs.	Exp.	Obs.	Exp.
Southeast Asia	Bangladesh, 2010	1.2	1.4	0.5	0.4	0.4	0.3	0.1	0.1	—	—	—	—	—	—	—	—
	Bangladesh, 2012	1.0	1.1	1.0	1.6	0.7	0.4	0.0	0.3	1.1	0.6	—	—	—	—	—	—
	Cambodia, 2014	0.0	0.0	0.0	0.0	0.0	0.0	0.0	0.0	0.0	0.0	0.0	0.0	0.0	0.0	0.0	0.0
	Laos, 2006	0.0	0.3	0.0	0.2	0.0	0.2	—	—	—	—	—	—	—	—	—	—
	Mongolia, 2006	1.3	0.0	9.7	9.9	4.0	4.7	2.6	3.0	8.0	8.4	1.4	1.7	6.5	7.0	—	—
	PNG, 2005	1.5	2.3	0.4	0.6	—	—	0.4	0.6	—	—	—	—	—	—	—	—
	Philippines, 2011	0.6	0.8	0.8	0.7	0.8	0.7	0.0	0.0	—	—	—	—	—	—	—	—
	Vietnam, 2010	0.0	0.3	2.4	2.8	1.1	0.7	0.0	0.2	1.4	2.1	0.0	0.1	0.3	0.8	—	—
Africa	Cote d'Ivoire, 2007	3.7	3.8	2.6	2.2	2.3	2.1	0.2	0.2	—	—	—	—	—	—	—	—
	Cameroon, 2009	1.7	1.8	2.8	2.7	0.8	1.2	0.6	0.3	1.9	2.0	0.7	0.3	—	—	1.0	0.6
	Kenya, 2007	3.5	3.1	3.9*	3.5	3.8*	3.4	0.2	0.3	—	—	—	—	—	—	—	—
	Kenya, 2010	3.3	3.2	2.5	2.5	2.5	2.4	0.0	0.4	—	—	—	—	—	—	—	—
	Liberia, 2011	1.2	1.3	1.1	1.2	1.1	1.2	0.0	0.1	—	—	—	—	—	—	—	—
	Malawi, 2016	2.6*	1.4	3.3	3.0	1.9*	1.0	0.5	0.4	2.3	2.8	—	—	—	—	—	—
Americas	Colombia, 2010	0.6	0.5	2.7	2.3	0.8	0.5	0.5	0.6	2.0	1.7	—	—	—	—	—	—
	Ecuador, 2012	1.2*	1.8	2.7	2.9	1.0	0.9	1.2	1.1	1.3	1.9	0.0	0.1	—	—	—	—
	Mexico, 2006	1.0	1.5	4.2*	3.4	3.4	2.6	—	—	3.0	2.0	0.6	0.4	—	—	0.0	0.2
	Mexico, 2012	1.5	1.5	2.3	2.1	1.8	1.5	0.6	0.6	—	—	0.2	0.0	—	—	0.0	0.0
	Nicaragua, 2005	1.2	1.3	1.5	1.4	3.0	2.3	0.1	0.0	—	—	—	—	—	—	—	—
	USA, 2006	0.1	0.2	2.2*	1.4	2.2*	1.2	—	—	—	—	0.2	0.2	0.1	0.1	0.0	0.0
Europe/Eastern Mediterranean	Afghanistan, 2013	1.9	2.9	6.1	5.4	2.3	1.7	3.2	2.8	1.6	1.7	—	—	4.3	3.1	—	—
	Azerbaijan, 2013	3.5	4.0	4.8	4.6	2.6	3.1	0.8	0.9	2.2*	1.2	—	—	—	—	—	—
	Georgia, 2009	5.0	4.5	0.1	0.0	0.1	0.0	—	—	—	—	—	—	—	—	—	—
	Pakistan, 2011	2.8	2.8	4.0	4.0	2.4	2.3	2.7*	2.3	2.2	2.2	—	—	0.9	0.8	—	—

1 Values are percentages. Surveys in descending order of OWOB prevalence within geographic groups. Asterisks indicate significant differences between Obs. and Exp. prevalence estimates calculated using the Rao–Scott modified chi-square test for independence of OWOB and each condition, except for Cambodia 2014, Laos 2006, and Mongolia 2006. Mongolia 2006 did not use a complex survey design, so the chi-square test was used. *P* values could not be computed for Laos 2006 because the observed DBM frequency was 0. No results are shown for Cambodia because the prevalence of OWOB was 0. Exp. prevalence of DBM was calculated by multiplying the prevalence of OWOB (BMI-for-age *z* score > 2 SD) by the prevalence of each undernutrition component of the DBM (anemia, MDI > 0, or individual micronutrient deficiencies). Iron deficiency was defined as inflammation-adjusted ferritin < 12 µg/L. Vitamin A deficiency was defined as inflammation-adjusted retinol-binding protein or retinol < 0.7 μmol/L. Zinc deficiency was defined as plasma or serum zinc < 650 µg/L for nonfasting children in the morning or <570 µg/L for nonfasting children in the afternoon/evening. Zinc concentrations were adjusted for inflammation in Afghanistan, Azerbaijan, Cambodia, Cameroon, and Ecuador. Vitamin D deficiency was defined as 25-hydroxyvitamin D < 30 nmol/L. Folate deficiency was defined as RBC folate < 226.5 nmol/L (if measured using the microbiologic assay) or serum folate < 6.8 nmol/L. Vitamin B-12 deficiency was defined as serum or plasma B-12 < 150 pmol/L. MDI was defined as the number of micronutrient biomarkers with values below the threshold used to define deficiency. DBM, double burden of malnutrition; Exp., expected; MDI, Micronutrient Deficiency Index; Obs., observed; OWOB, overweight or obesity.

### Independence of DBM components

In the majority of surveys, there was no significant association between OWOB and micronutrient deficiency or anemia (Table 3). OWOB and micronutrient deficiency (MDI > 0) were positively associated in 4 surveys; among these, the differences between the expected and observed prevalence of DBM were <1 percentage point. In these 4 surveys, the prevalence of MDI > 0 among OWOB children compared with children with BAZ ≤ 2 SD was 85.0% compared with 74.2% in Kenya 2007, 68.9% compared with 58.2% in Colombia, 61.7% compared with 48.8% in Mexico 2006, and 23.0% compared with 13.4% in the USA (**Supplemental Table 3**). In all, OWOB and iron deficiency were associated in 3 of 23 surveys for which serum or plasma ferritin was measured. With 2 exceptions (vitamin A in Pakistan and zinc in Azerbaijan), we did not observe associations between other individual micronutrient deficiencies and OWOB.

OWOB and anemia were not associated in all but 2 surveys. However, in Malawi OWOB children were more likely to have anemia (57.0% compared with 29.5% anemia among children with BAZ > 2 and ≤2 SD, respectively) (Supplemental Table 3), and children with OWOB were less likely to have anemia than were children with BAZ ≤ 2 SD in Ecuador (16.4% compared with 25.3%) and Mexico 2006 (13.6% compared with 21.2%).

### Predictors of DBM

**FIGURE 2:**
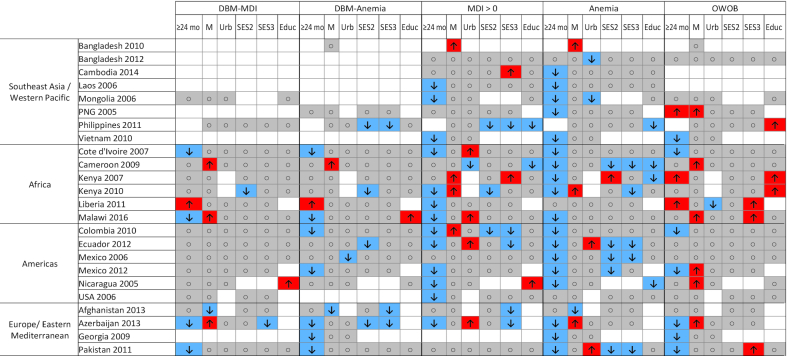
Patterns of associations between the DBM, micronutrient deficiency, anemia, and OWOB. Blue cells with the “down” arrow indicate smaller odds of the outcome (*P* < 0.05), red cells with the “up” arrow indicate greater odds of the outcome (*P* < 0.05), gray cells with the circle indicate no significant association (*P* ≥ 0.05), and white cells indicate that the relation was not assessed owing to missing data. Results reflect logistic regression analysis (SAS proc surveylogistic) with all predictor variables included in each model. Analysis of predictors of DBM-MDI and DBM-Anemia was not conducted for surveys with *n* < 10 cases of the outcome (DBM-MDI: Bangladesh 2010, Bangladesh 2012, Cambodia, Georgia, Laos, PNG, Vietnam; DBM-Anemia: Bangladesh 2012, Cambodia, Laos, Mongolia, USA, Vietnam). DBM, double burden of malnutrition; DBM-Anemia, double burden of malnutrition, defined as overweight/obesity and anemia; DBM-MDI, double burden of malnutrition, defined as overweight/obesity and ≥1 micronutrient deficiency; Educ, secondary or higher caregiver or head of household education (reference: no or primary education); M, male sex (reference: female sex); MDI, Micronutrient Deficiency Index; OWOB, overweight or obesity; PNG, Papua New Guinea; SES2, medium socioeconomic status (reference: low socioeconomic status); SES3, high socioeconomic status (reference: low socioeconomic status); Urb, urban household location (reference: rural household location); ≥24 mo, child age 24–59 mo (reference: age 6 to <24 mo).

Generally, child sex, urban or rural household location, household SES, and respondent education were not found to be associated with DBM-MDI or DBM-Anemia (**Figure 2**; **Supplemental Tables 4–7**). The most consistent predictor of DBM was child age: children <24 mo of age were more likely to experience DBM-Anemia than were those ≥24 mo of age, in 7 of the 19 surveys for which the comparison could be conducted. Similarly, younger children were more likely to have anemia (19 of 23 surveys) and MDI > 0 (13 of 22 surveys) (**Supplemental Tables 8**, **9**). However, DBM-MDI was more likely among younger children in only 4 of 19 surveys (and was less likely among younger children in Liberia), whereas associations between child age and OWOB were mixed, with OWOB more common among younger children in 7 surveys and among older children in 3 surveys (Figure 2; **Supplemental Table 10**). Male children were more likely to have OWOB than were female children in 6 of 24 surveys (Figure 2). In the majority of surveys, no association was observed between DBM or its components and SES or respondent education level. In cases where the associations were significant, children in households with high SES and high caregiver or head of household education were more likely to have OWOB and less likely to have anemia (Figure 2). In almost one-third of surveys (5 of 16), individuals with lower SES were more likely to have DBM-Anemia; similarly, odds of anemia were greater for children in low-SES households in 6 of 19 surveys (Figure 2).

## Discussion

We have reported the prevalence and correlates of intra-individual DBM and its components among preschool children in 24 surveys in diverse geographic locations. The relatively low prevalence of DBM (<10%) appears to be constrained by the low prevalence of OWOB among children (<10% in all but 2 surveys and close to 0 in several surveys), rather than micronutrient deficiencies or anemia (>20% in most surveys). If child overweight continues to increase as projected (1), we might expect an increase in prevalence of DBM among children at both the individual and population levels.

This analysis provides a detailed multicountry characterization of DBM among children at the individual level using multiple micronutrient biomarkers. Other studies that have examined DBM among preschool children have predominantly used anthropometric indicators to define both over- and under-nutrition (31,32). At the population level, DBM is often characterized as coexistence of OWOB children or adults and stunted or underweight children (33, 34); at the individual level, studies have reported the co-occurrence of OWOB and stunting among children (14). Other DBM analyses have used anemia as a measure of undernutrition (16), with the advantage that these data are widely available through Demographic and Health Surveys. However, the etiology of anemia is multifactorial and includes many nonnutritional causes, such as genetic hemoglobin disorders and inflammation (35), so anemia is not specific to undernutrition. Although the prevalence of DBM among children in this analysis was similar when defined with micronutrient biomarkers or anemia, this is likely explained by the low prevalence of OWOB. We also observed variability in the prevalence of micronutrient deficiencies compared with anemia across surveys, and anemia and the MDI were not correlated at the survey level. Thus, anemia may be useful for monitoring population health, but has important limitations as an indicator of undernutrition or micronutrient deficiency. Few studies have used micronutrient deficiencies in their definition of DBM; these have been of limited geographic scope or constrained to single micronutrient deficiencies (17, 36).

In the surveys we examined, child OWOB was largely independent of undernutrition measured as individual micronutrient deficiencies, the MDI, or anemia. In particular, we did not observe consistent associations between OWOB and iron deficiency, despite evidence from previous studies that individuals with OWOB are at risk of iron deficiency (19). Our observations may be explained in part by the low prevalence of child overweight (<5% in half the surveys) and consequent low statistical power to detect associations with OWOB (almost half of the surveys had sample sizes < 1000); if so, significant associations might be detected with a greater sample size. An alternate explanation would be that evidence of metabolic interactions between OWOB and micronutrient deficiencies or anemia may be more apparent as mean BAZ increases. However, analyses of data for women of reproductive age with greater prevalence of OWOB (37) also reported limited evidence for associations between OWOB and micronutrient deficiencies or anemia in population-based surveys, suggesting that low prevalence of overweight does not entirely explain the lack of association. In this epidemiological analysis, other individual- or household-level factors, such as differences in dietary patterns and exposure to infections, may emerge as more prominent than obesity-related alterations in micronutrient metabolism. Observed associations should be interpreted in light of the 5% level of significance, where under the null hypothesis of no relations, 1 in 20 calculations have spurious positive findings. Nevertheless, the positive associations observed between OWOB and micronutrient deficiencies in several surveys (particularly in Mexico and the USA, where prevalence of overweight among preschool children is high at ∼7–9% BAZ > 2 SD in both countries) merit further investigation. In all countries, leveraging representative data on micronutrient deficiencies, anemia, and OWOB is an important step toward designing and managing nutrition programs that aim to address multiple forms of malnutrition.

The prevalence of DBM was not consistently associated with child sex, urban or rural location, household SES, or education. As aforementioned, the lack of association may reflect in part low statistical power because the prevalence estimates of DBM were low. Harmonization of predictor variable definitions, such as for SES, is necessary for such an analysis but results in loss of information that could be important for an individual country. However, given that OWOB and indicators of undernutrition (e.g., MDI and anemia) were not found to be statistically dependent in most surveys, it is not surprising that few characteristics would predict their combination. The lower prevalence of DBM-MDI and particularly DBM-Anemia among older children (≥24 mo) observed in some surveys may reflect the fact that anemia and micronutrient deficiencies were also more common among younger children (<24 mo). The regression model results do not support any obvious shared causes underlying both micronutrient deficiencies and OWOB, nor is there a clear pattern of “opposite” relations; for example, one might hypothesize that higher SES would be associated with greater odds of obesity and lower odds of micronutrient deficiency, but that was not apparent. More detailed country-level analyses may reveal the importance of individual- and household-level factors that were not available for this analysis.

A limitation of this analysis is that the MDI is a crude measure of micronutrient deficiency burden, which does not take into account severity of deficiency and combines data from micronutrients with disparate biological roles and food sources. Thus, patterns for individual nutrients may be obscured by creating a multiple-micronutrient index. We chose to examine the associations between OWOB and individual micronutrient deficiencies to determine if there was substantial masking of single micronutrient associations when collapsed into the index. However, there were very few cases in which OWOB was associated with individual micronutrient deficiencies but not with the MDI (Azerbaijan, Malawi, and Pakistan), and the reverse was found in Colombia and Ecuador. Another limitation of the index is that it depends on the number and type of micronutrients measured, but some surveys included few micronutrient biomarkers. Lastly, certain biomarkers are not intended for use at the individual level (e.g., serum zinc and retinol). The potential for misclassification of individual micronutrient status would tend to bias results toward a lack of association with other measures. Nevertheless, the MDI offers a summary measure of multiple micronutrient deficiencies which may be improved upon in future work.

Another limitation of this cross-sectional analysis is that we could not describe manifestations of DBM across the life course. We focused on children who experience OWOB before age 5 y and, in turn, are likely to experience OWOB later in life. Yet, the results do not capture the situation of children who are malnourished in early life but subsequently develop OWOB; these individuals may be at particularly high risk of developing metabolic disease later in life (38). Because micronutrient deficiencies and anemia may be present in situations of caloric deficit or excess, further research on this topic may help clarify the short- and long-term implications of various manifestations of the DBM.

This examination of the patterns of the DBM and its components in diverse settings has several implications for nutrition programs. First, both micronutrient deficiencies and overweight are present among preschool children in many countries [with the exception of several Southeast Asian countries with low prevalence of overweight, although global trends suggest that these settings are also experiencing increases in average BMI (1)]. Coexistence of these conditions at the national level will further stress resource-constrained health care systems, and coexistence at the individual level is likely to compound the adverse health consequences across the life course. However, the failure to detect an association between OWOB and micronutrient deficiencies or anemia in most surveys suggests that targeting of specific interventions for *individuals* afflicted by the DBM may not be necessary. Even if such targeting were employed to reach individuals at risk of greater health consequences due to overlapping OWOB and undernutrition, our analysis did not identify characteristics that could reliably predict individuals at risk of the DBM. Instead, at the population level, our study suggests that public health efforts focused on addressing the component problems (namely, micronutrient deficiencies, anemia, and OWOB) and their respective causes may be a more efficient strategy. Research on the etiology of these conditions in different settings and on the efficacy and effectiveness of interventions could improve efforts to effectively address multiple forms of malnutrition in populations.

## Supplementary Material

nqaa101_Supplemental_File

## Notes

Supported by the Bill & Melinda Gates Foundation, CDC, Eunice Kennedy Shriver National Institute of Child Health and Human Development, HarvestPlus, and the United States Agency for International Development.

Supplemental Tables 1–10 are available from the “Supplementary data” link in the online posting of the article and from the same link in the online table of contents at https://academic.oup.com/ajcn/.

Data described in the article, code book, and analytic code will be made available upon request pending approval from the BRINDA steering committee and country representatives.

Publication costs for this supplement were defrayed in part by the payment of page charges by the Bill & Melinda Gates Foundation. The Guest Editor for this supplement was Daniel Roth, who has no relevant disclosures. The Supplement Coordinators for the supplement publication were Parminder S Suchdev and Melissa F Young, Emory University, Atlanta, GA 30322, USA. Supplement Coordinator disclosure: PSS receives salary support from the CDC; MFY: no conflicts to disclose. The opinions expressed in this publication are those of the authors and are not attributable to the sponsors or the publisher, Editor, or Editorial Board of *The American Journal of Clinical Nutrition*.

Abbreviations used: AGP, α-1-acid glycoprotein; BAZ, BMI-for-age *z* score; BRINDA, Biomarkers Reflecting Inflammation and Nutritional Determinants of Anemia; CRP, C-reactive protein; DBM, double burden of malnutrition; DBM-Anemia, double burden of malnutrition, defined using anemia as the indicator of undernutrition; DBM-MDI, double burden of malnutrition, defined using micronutrient deficiency as the indicator of undernutrition; MDI, Micronutrient Deficiency Index; NCD, noncommunicable disease; OWOB, overweight or obesity; RBCF, red blood cell folate; SES, socioeconomic status; WHZ, weight-for-height *z* score; 25(OH)D, 25-hydroxyvitamin D.
