# Supplementary material for: Intraindividual double burden of overweight and micronutrient deficiencies or anemia among preschool children
Source: Am J Clin Nutr. 2020 Aug 4;112(Suppl 1):478S–487S. doi: 10.1093/ajcn/nqaa101 (PMC7396269; doi:10.1093/ajcn/nqaa101)
Supplement: nqaa101_Supplemental_File [file nqaa101_supplemental_file.docx]

**Intra-individual double burden of overweight and micronutrient deficiencies or anemia among preschool children**

Engle-Stone et al.

**Online Supplementary Material**

**Supplementary Table 1.** Surveys of pre-school children included in the analysis, and associated references (Note: up-to-date information is also included on the project website: <https://brinda-nutrition.org/about-us/brinda-countries/>)

| **No.** | **Survey** | **Year** | **Survey Name** | **General References** | **Links** |
| --- | --- | --- | --- | --- | --- |
| 1 | Afghanistan | 2013 | National Nutrition Survey Afghanistan 2013 | UNICEF. (2014). National nutrition survey: Afghanistan (2013). *Kabul: United Nations ChildrenLs Fund.* | <https://reliefweb.int/sites/reliefweb.int/files/resources/Report%20NNS%20Afghanistan%202013%20%28July%2026-14%29.pdf> |
| 2 | Azerbaijan | 2013 | AZERBAIJAN NUTRITION SURVEY (AzNS), 2013 | UNICEF. (2013). Azerbaijan Nutrition Survey (AzNS), 2013. | <http://groundworkhealth.org/wp-content/uploads/2015/06/UNICEF-2013_Azerbaijan-National-Nutrition-Survey_report_eng_compressed.pdf> |
| 3 | Bangladesh | 2010 | Bangladesh MNP Baseline Survey | N/A | Unavailable; contact authors |
| 4 | Bangladesh | 2012 | National Micronutrients Status Survey 2011-12 | ICDDR B, UNICEF (Bangladesh), GAIN, Institute of Public Health and Nutrition. (2013). National micronutrients status survey 2011–12. | <https://www.gainhealth.org/sites/default/files/publications/documents/bangladesh-national-micronutrient-survey-final-report-2013.pdf> |
| 5 | Cambodia | 2014 | Cambodia Demographic and Health Survey 2014 (CDHS-2014) | Kosal, S., Satia, C., Kheam, T., Chinda, P., Mondol, L., Phirun, L., ... & Kishor, S. (2015). Cambodia Demographic and Health Survey 2014. *Phnom Penh: National Institute of Statistics, Directorate General for Health, and ICF International*. | <https://dhsprogram.com/pubs/pdf/FR312/FR312.pdf> |
| 6 | Cameroon | 2009 | a nationally representative, cross-sectional, multistage cluster survey of women (aged 15–49 y) and preschool children (aged 12–59 mo) in the Republic of Cameroon | Engle-Stone, R., Ndjebayi, A. O., Nankap, M., & Brown, K. H. (2012). Consumption of potentially fortifiable foods by women and young children varies by ecological zone and socio-economic status in Cameroon. *The Journal of nutrition*, *142*(3), 555-565. | <https://pdfs.semanticscholar.org/5afc/5cf128b081ddca35bafffc76a578a159cd3c.pdf?_ga=2.35494305.1076716055.1582054548-680600078.1582054548> |
| 7 | Cote d'Ivoire | 2007 | a nationally representative cross-sectional survey was conducted in Coˆte d’Ivoire in July/August 2007 | Rohner, F., Tschannen, A. B., Northrop-Clewes, C., Kouassi-Gohou, V., Bosso, P. E., & Mascie-Taylor, C. N. (2012). Comparison of a possession score and a poverty index in predicting anaemia and undernutrition in pre-school children and women of reproductive age in rural and urban Cote d'Ivoire. *Public health nutrition*, *15*(9), 1620-1629. | <https://doc.rero.ch/record/298729/files/S1368980012002819.pdf> |
| 8 | Colombia | 2010 | 2010 Encuesta Nacional de la Situaci ón Nutricional en Colombia (ENSIN) | de Lleras, C. D. L. F. (2010). Encuesta Nacional de la situación nutricional en Colombia 2010 [National Survey of the nutritional situation in Colombia 2010]. | <https://www.minsalud.gov.co/sites/rid/Lists/BibliotecaDigital/RIDE/VS/ED/GCFI/Base%20de%20datos%20ENSIN%20-%20Protocolo%20Ensin%202010.pdf> |
| 9 | Ecuador | 2012 | 2012 Ecuadorian National Health and Nutrition Survey (ENSANUT-ECU) | Freire, W. B., Belmont, P., López-Cevallos, D. F., & Waters, W. F. (2015). Ecuador's National Health and Nutrition Survey: objectives, design, and methods. *Annals of epidemiology*, *25*(11), 877-878. | <https://europepmc.org/article/med/26386743> |
| 10 | Georgia | 2009 | Georgia National Nutrition Survey 2009 | UNICEF. (2010). Report of the Georgia National Nutrition Survey, 2009. | Unavailable; contact authors |
| 11 | Kenya | 2007 | Nyando Integrated Child Health and Education Project (NICHE) - Baseline | Centers for Disease Control and Prevention (CDC. (2007). Baseline data from the Nyando Integrated Child Health and Education Project--Kenya, 2007. *MMWR. Morbidity and mortality weekly report*, *56*(42), 1109. | <https://www.cdc.gov/mmwR/pdf/wk/mm56e1022.pdf> |
| 12 | Kenya | 2010 | Nyando Integrated Child Health and Education Project (NICHE)- FU2010 | Foote, E. M., Sullivan, K. M., Ruth, L. J., Oremo, J., Sadumah, I., Williams, T. N., & Suchdev, P. S. (2013). Determinants of anemia among preschool children in rural, western Kenya. *The American journal of tropical medicine and hygiene*, *88*(4), 757-764. | <https://www.ncbi.nlm.nih.gov/pmc/articles/PMC3617865/pdf/tropmed-88-757.pdf> |
| 13 | Laos | 2006 | National Maternal and Child Nutrition Survey (MICS3-NNS). The Lao PDR | Laos Service national de la statistique, Laos Hygiene and Prevention Department. National Maternal and Child Nutrition Survey (MICS3-NNS) Report, the Lao PDR, 2006: Final Report: Ministry of Planning and Investment, Department of Statistics, 2009. | Hardcopy available in Emory library. |
| 14 | Liberia | 2011 | Liberia National Micronutrient Survey 2011 | UNICEF. (2011). Liberia National Micronutrient Survey 2011—Selected Preliminary Findings. *UNICEF, Liberia Institute of Statistics: Monrovia, Liberia*. | <http://ghdx.healthdata.org/record/liberia-national-micronutrient-survey-2011> |
| 15 | Malawi | 2016 | The 2015-16 Malawi Micronutrient Survey (MNS) | NSONM, I. (2017). Malawi demographic and health survey 2015–16. *Zomba, Malawi, and Rockville, Maryland, USA*. | <https://dhsprogram.com/pubs/pdf/FR319/FR319.pdf> |
| 16 | Mexico | 2006 | the Mexican National Health and Nutrition Survey 2006 (ENSANUT 2006) / ENCUESTA NACIONAL DE SALUD Y NUTRICIÓN 2006 | Abúndez, C. O., Cázares, G. N., Cordero, C. J. F. R., Zetina, D. A. D., Angona, S. R., de Voghel Gutiérrez, S., ... & Rivera-Dommarco, J. (2006). Encuesta nacional de salud y nutrición 2006 [National Health and Nutrition Survey 2006]. *Instituto Nacional de Salud Pública*. | <https://s3.amazonaws.com/academia.edu.documents/7686081/ensanut2006.pdf?response-content-disposition=inline%3B%20filename%3DEncuesta_Nacional_de_Salud_y_Nutricion_2.pdf&X-Amz-Algorithm=AWS4-HMAC-SHA256&X-Amz-Credential=AKIAIWOWYYGZ2Y53UL3A%2F20200219%2Fus-east-1%2Fs3%2Faws4_request&X-Amz-Date=20200219T212209Z&X-Amz-Expires=3600&X-Amz-SignedHeaders=host&X-Amz-Signature=d110fa64849ca1251aa8f3e872b435020bd1d56c00a83ba822e67a57f0386d55> |
| 17 | Mexico | 2012 | the Mexican National Health and Nutrition Survey 2012 (ENSANUT 2012) / ENCUESTA NACIONAL DE SALUD Y NUTRICIÓN 2012 | Gutierrez, J. P., Rivera-Dommarco, J., Shamah-Levy, T., Villalpando-Hernández, S., Franco, A., Cuevas-Nasu, L., ... & Hernández-Ávila, M. (2012). Encuesta nacional de salud y nutrición 2012 [National Health and Nutrition Survey 2012]. *Resultados Nacionales. Cuernavaca, México: Instituto Nacional de Salud Pública*, *1*(1.48). | <https://ensanut.insp.mx/encuestas/ensanut2012/doctos/informes/ENSANUT2012ResultadosNacionales.pdf> |
| 18 | Mongolia | 2006 | Nutritional Status of Mongolian Children and Women 2004- 3rd National Nutrition Survey. | Lander, R. L., Enkhjargal, T., Batjargal, J., Bailey, K. B., Diouf, S., Green, T. J., ... & Gibson, R. S. (2008). Multiple micronutrient deficiencies persist during early childhood in Mongolia. *Asia Pacific Journal of Clinical Nutrition*, *17*(3), 429-440. | <https://pdfs.semanticscholar.org/4845/9a43767477b52cb8dbb60cf05a3635f9d61d.pdf> |
| 19 | Nicaragua | 2005 | National Micronutrient Survey / Sistema Integrado de Vigilancia de Intervenciones Nutricionales (SIVIN) | Gurdián, M., Kontorovsky, I., Alvarado, E., Ramírez, S., & Hernández, R. (2005). Sistema Integrado de Vigilancia de Intervenciones Nutricionales (SIVIN) [Integrated Nutrition Intervention Monitoring System (SIVIN)]. *Informe de Progreso. Managua, Nicaragua: Ministerio de Salud*. | <http://www.incap.int/sisvan/index.php/es/areas-tematicas/metodologias-de-apoyo/sistema-integrado-de-vigilancia-de-intervenciones-nutricionales-sivin> |
| 20 | Papua New Guinea | 2005 | National Micronutrient Survey, Papua New Guinea, 2005 (PNG NNS 2005) | National Department of Health, U. P., University of Papua New Guinea, US Centre for Disease Control. (2011). Papua New Guinea National Nutrition Survey, 2005 (PNG NNS 2005). *Pacific Journal of Medical Sciences Volume 8, No. 2*. | [https://www.pacjmedsci.com//PJMS%20Vol%208%20No%202%20special%20issue%20May%202011.pdf](https://www.pacjmedsci.com/PJMS%20Vol%208%20No%202%20special%20issue%20May%202011.pdf) |
| 21 | Pakistan | 2011 | National Nutrition Survey Pakistan 2011 | Bhutta, Z. A., Soofi, S. B., Zaidi, S. S. H., & Habib, A. (2011). Pakistan National Nutrition Survey, 2011. | <https://ecommons.aku.edu/cgi/viewcontent.cgi?article=1262&context=pakistan_fhs_mc_women_childhealth_paediatr> |
| 22 | Philippines | 2011 | A Baseline Survey and Formative Research Ensuring Food Security and Nutrition among Children 0-23 Months of Age in the Philippines | Saniel, O. P., Rabuco, L. B., & Lebanan, M. A. O. Baseline Survey and Formative Research Ensuring Food Security and Nutrition among Children 0-23 Months of Age in the Philippines. | <https://www.gainhealth.org/sites/default/files/publications/documents/baseline-survey-and-formative-research-ensuring-food-security-and-nutrition-philippines.pdf> |
| 23 | United States | 2006 | National Health and Nutrition Examination Surveys (NHANES) 2003-2006 | Centers for Disease Control and Prevention. (2012). Second national report on biochemical indicators of diet and nutrition in the US population. *Atlanta (GA): CDC*. | <https://www.cdc.gov/nutritionreport/pdf/Nutrition_Book_complete508_final.pdf> |
| 24 | Vietnam | 2010 | Micronutrient study (MNS) 2010 | Laillou, A., Van Pham, T., Tran, N. T., Le, H. T., Wieringa, F., Rohner, F., ... & Berger, J. (2012). Micronutrient deficits are still public health issues among women and young children in Vietnam. *PloS one*, *7*(4). | <https://journals.plos.org/plosone/article/file?type=printable&id=10.1371/journal.pone.0034906> |

**Supplementary Table 2**. Biomarker availability and lab methods by survey^1^

| **Country (Year)** | **Micronutrient biomarkers** | | | | | | | | **Hemoglobin** | | | |
| --- | --- | --- | --- | --- | --- | --- | --- | --- | --- | --- | --- | --- |
|  | **Iron** | | **Vitamin A** | | **Zinc** | **Vitamin B12** | **Folate** | **Vitamin D** | **Method** | **Blood** | **Adjusted for altitude** | **Adjusted for smoking** |
|  | **Biomarker** | **Method** | **Biomarker** | **Method** |  |  |  |  |  |  |  |  |
| Afghanistan (2013) | Ferritin | TIA | Retinol | HPLC | AAS | Not measured | Not measured | CLIA | HemoCue Hb 201 | Venous | Yes | No |
| Azerbaijan (2013) | Ferritin | Sandwich ELISA | RBP | Sandwich ELISA | ICP-OES | Microbiological assay | Microbiological assay | Not measured | HemoCue Hb 201+ | Capillary | Yes | Yes^2^ |
| Bangladesh (2010) | Ferritin | Sandwich ELISA | RBP | Sandwich ELISA | Not measured | Not measured | Not measured | Not measured | HemoCue Hb 201+ | Capillary | No | No |
| Bangladesh (2012) | Ferritin | Sandwich ELISA | Retinol | HPLC | AAS | Not measured | Not measured | Not measured | HemoCue Hb 301 | Venous | No | No |
| Cambodia (2014) | Ferritin | Sandwich ELISA | RBP | Sandwich ELISA | AAS | ECLIA | ECLIA | ECLIA | HemoCue Hb 301 | Capillary | No | Yes |
| Cameroon (2009) | Ferritin | Sandwich ELISA | RBP | Sandwich ELISA | AAS | Radioassay | Radioassay | Not measured | HemoCue Hb 201+ | Venous | No | No |
| Colombia (2010) | Ferritin | CLIA | Retinol | HPLC | AAS | CLIA | Not measured | Not measured | HemoCue | Capillary | Yes | Yes |
| Côte d’Ivoire (2007) | Ferritin | Sandwich ELISA | RBP | Sandwich ELISA | Not measured | Not measured | Not measured | Not measured | HemoCue Hb 201+ | Venous | No | No |
| Ecuador (2012) | Ferritin | CLIA | Retinol | HPLC | AAS | CLIA | CLIA | Not measured | Sysmex XE-2100 | Venous | Yes | Yes |
| Georgia (2009) | Ferritin | Turbidimetry | Retinol/RBP | Not measured | Not measured | Not measured | Not measured | Not measured | HumaMeter | Capillary | Yes | Yes |
| Kenya (2007) | Ferritin | Sandwich ELISA | RBP | Sandwich ELISA | Not measured | Not measured | Not measured | Not measured | HemoCue Hb-B | Capillary | No | No |
| Kenya (2010) | Ferritin | Sandwich ELISA | RBP | Sandwich ELISA | Not measured | Not measured | Not measured | Not measured | HemoCue Hb-B | Capillary | No | No |
| Laos (2006) | Ferritin | Sandwich ELISA | Retinol/RBP | Not measured | Not measured | Not measured | Not measured | Not measured | HemoCue Hb-B | Capillary | Yes | No |
| Liberia (2011) | Ferritin | Sandwich ELISA | RBP | Sandwich ELISA | Not measured | Not measured | Not measured | Not measured | HemoCue Hb 201+ | Capillary | No | No |
| Malawi (2016) | Ferritin | Sandwich ELISA | RBP | Sandwich ELISA | AAS | Not measured | Not measured | Not measured | HemoCue Hb 301 | Venous | Yes | Yes^2^ |
| Mexico (2006) | Ferritin | Immunoassay | Retinol/RBP | Not measured | ICP-OES | EIA | EIA | Not measured | HemoCue | Capillary | Yes | Yes |
| Mexico (2012) | Ferritin | CMIA | Retinol | HPLC | Not measured | CMIA | CMIA | Not measured | HemoCue | Capillary | Yes | Yes |
| Mongolia (2006) | Ferritin | MEIA | Retinol | HPLC | AAS | Not measured | Microbiological assay | RIA | HemoCue | Capillary | Yes | No |
| Nicaragua (2005) | Ferritin | Immunoassay | Retinol | HPLC | Not measured | Not measured | Not measured | Not measured | HemoCue Hb-B | Capillary | No | No |
| Pakistan (2011) | Ferritin | Turbidimetry | Retinol | HPLC | AAS | Not measured | Not measured | CLIA | HemoCue Hb 201+ | Venous | No | No |
| Papua New Guinea (2005) | sTfR | Sandwich ELISA | RBP | Sandwich ELISA | Not measured | Not measured | Not measured | Not measured | HemoCue Hb 201+ | Capillary | Yes | Yes |
| Philippines (2011) | Ferritin | Sandwich ELISA | RBP | Sandwich ELISA | Not measured | Not measured | Not measured | Not measured | HemoCue Hb 201+ | Capillary | No | No |
| United States (2006) | Ferritin | IRMA | Retinol | Reversed-phase HPLC | Not measured | Radioassay | Radioassay | RIA | HMX Hematology Analyzer | Venous | No | Yes |
| Vietnam (2010) | Ferritin | ELISA | Retinol | Reversed-phase HPLC | AAS | Not measured | Microbiological assay | HPLC | HemoCue Hb 301 | Venous | No | No |

^1^ The VitMin Laboratory analyzed all samples in which the sandwich ELISA technique was used except Bangladesh 2012. AAS, atomic absorption spectrometer; CLIA, chemiluminescent immunoassay; CMIA, chemiluminescent microparticle immunoassay; ECLIA, electrochemiluminescence immunoassay; EIA, enzyme immunoassay; ELISA, enzyme-linked immunosorbent assay; HPLC, high performance liquid chromatography; ICP-OES, inductively coupled plasma-optical emission spectrometry; IRMA, immunoradiometric assay; MEIA, microparticle enzyme immunoassay; RBP, retinol binding protein; RIA, radioimmunoassay; sTfR, soluble transferrin receptor; TIA, turbidimetric immunoassay.

^2^ All respondents in Azerbaijan reported no smoking, only 1 respondent in Malawi reported smoking.

**Supplementary Table 3. Prevalence of anemia, MDI > 0, and micronutrient deficiencies among children with overweight or obesity (BAZ > 2 SD) compared to children with BAZ ≤ 2 SD.**

| Country |  | Anemia, % | MDI > 0, % | Iron, % | Vitamin A, % | Zinc, % | Folate, % | Vitamin D, % | Vitamin B12, % |
| --- | --- | --- | --- | --- | --- | --- | --- | --- | --- |
| Bangladesh 2010 | OW/OB | 68.0  (46.7, 89.3) | 28.0  (5.0, 51.0) | 24.0  (4.6, 43.4) | 4.0  (0, 12.6) | 77.5  (9.6, 100.0) | -- | -- | -- |
|  | BAZ ≤ 2 | 83.7  (81.4, 86.0) | 20.9  (18.1, 23.7) | 15.8  (13.1, 18.5) | 6.2  (4.3, 8.2) | 46.9  (35.5, 58.2) | -- | -- | -- |
| Bangladesh 2012 | OW/OB | 27.6  (0, 69.2) | 28.0 (0, 70.5) | 20.5  (0, 53.2) | -- | -- | -- | -- | -- |
|  | BAZ ≤ 2 | 32.0  (23.1, 40.9) | 45.8  (37.5, 54.1) | 11.3  (6.0, 16.6) | 10.3  (4.4, 16.2) | -- | -- | -- | -- |
| Cambodia 2014 | OW/OB | -- | -- | -- | -- | -- | -- | -- | -- |
|  | BAZ ≤ 2 | 53.3  (47.4, 59.2) | 61.0  (52.4, 69.5) | 5.4  (2.9, 7.9) | -- | 62.8  (53.9, 71.7) | 6.0  (3.3, 8.8) | 4.7  (1.3, 8.0) | 2.1  (0.6, 3.6) |
| Laos 2006 | OW/OB | -- | -- | -- | -- | -- | -- | -- | -- |
|  | BAZ ≤ 2 | 40.8  (31.3, 50.3) | 25.8  (21.0, 30.6) | 25.8  (21.0, 30.6) | -- | -- | -- | -- | -- |
| PNG 2005 | OW/OB | 31.1  (8.8, 53.3) | 8.4  (0, 19.0) | -- | 8.4  (0, 19.0) | -- | -- | -- | -- |
|  | BAZ ≤ 2 | 49.0  (43.4, 54.7) | 11.7  (9.2, 14.3) | -- | 11.7  (9.2, 14.3) | -- | -- | -- | -- |
| Philippines 2011 | OW/OB | 32.0  (7.9, 56.0) | 38.1  (8.5, 67.7) | 38.1  (8.5, 67.7) | 0  (0, 0) | -- | -- | -- | -- |
|  | BAZ ≤ 2 | 42.1  (37.9, 46.2) | 35.3  (31.7, 38.9) | 34.9  (31.3, 38.4) | 1.0  (0.4, 1.5) | -- | -- | -- | -- |
| Vietnam 2010 | OW/OB | -- | 64.3  (31.7, 96.9) | 28.6  (0, 60.5) | -- | 35.7  (0, 72.3) | -- | 8.3  (8.3, 8.3) | -- |
|  | BAZ ≤ 2 | 7.8  (4.6, 11.1) | 73.8  (69.2, 78.4) | 18.1  (14.0, 22.3) | 5.9  (3.2, 8.5) | 57.3  (51.9, 62.8) | 4.2  (1.0, 7.4) | 21.3  (16.2, 26.5) | -- |
| Cote d’Ivoire 2007 | OW/OB | 68.9  (49.1, 88.8) | 48.2  (31.5, 64.9) | 43.7  (27.1, 60.2) | 4.5  (0, 13.8) | -- | -- | -- | -- |
|  | BAZ ≤ 2 | 72.0  (67.1, 77.0) | 40.6  (36.2, 45.1) | 38.7  (34.4, 42.9) | 3.0  (1.6, 4.4) | -- | -- | -- | -- |
| Cameroon 2009 | OW/OB | 51.6  (31.6, 71.6) | 79.2  (58.2, 100) | 24.1  (3.9, 44.3) | 18.0  (1.6, 34.4) | 54.2  (32.4, 76.0) | 17.5  (0, 42.9) | -- | 26.1  (0, 54.9) |
|  | BAZ ≤ 2 | 55.1  (50.0, 60.2) | 75.9  (72.3, 79.5) | 35.3  (30.9, 39.6) | 9.6  (7.2, 12.1) | 58.6  (53.7, 63.5) | 8.1  (4.6, 11.6) | -- | 14.9  (10.0, 19.8) |
| Kenya 2007 | OW/OB | 75.0  (60.0, 90.0) | 85.0  (75.5, 94.5) | 82.5  (72.3, 92.7) | 5.0  (0, 12.1) | -- | -- | -- | -- |
|  | BAZ ≤ 2 | 66.7  (62.7, 70.6) | 74.2  (70.7, 77.8) | 72.5  (68.8, 76.2) | 6.3  (4.3, 8.3) | -- | -- | -- | -- |
| Kenya 2010 | OW/OB | 75.7  (63.3, 88.1) | 56.8  (42.9, 70.7) | 56.8  (42.9, 70.7) | -- | -- | -- | -- | -- |
|  | BAZ ≤ 2 | 71.3  (67.6, 75.0) | 57.6  (53.8, 61.3) | 53.6  (49.6, 57.5) | 8.6  (6.9, 10.3) | -- | -- | -- | -- |
| Liberia 2011 | OW/OB | 54.0  (34.7, 73.2) | 48.1  (27.8, 68.5) | 46.4  (25.9, 66.9) | 1.7  (0, 5.3) | -- | -- | -- | -- |
|  | BAZ ≤ 2 | 59.6  (55.7, 63.6) | 53.4  (49.3, 57.4) | 51.2  (47.2, 55.2) | 5.4  (3.9, 7.0) | -- | -- | -- | -- |
| Malawi 2016 | OW/OB | 57.0  (41.6, 72.3) | 71.9  (55.9, 87.9) | 41.3  (23.2, 59.4) | 10.1  (1.6, 18.6) | 34.8  (18.5, 51.2) | -- | -- | -- |
|  | BAZ ≤ 2 | 29.5  (25.9, 33.1) | 63.8  (59.0, 68.7) | 20.9  (15.9, 26.0) | 7.8  (5.1, 10.4) | 49.7  (43.6, 55.8) | -- | -- | -- |
| Colombia 2010 | OW/OB | 16.4  (9.0, 23.8) | 68.9  (59.8, 78.0) | 21.3  (12.6, 30.0) | 12.8  (6.1, 19.6) | 53.4  (42.8, 64.1) | -- | -- | -- |
|  | BAZ ≤ 2 | 13.0  (11.5, 14.5) | 58.2  (56.0, 60.4) | 13.3  (11.8, 14.7) | 17.8  (16.1, 19.6) | 44.5  (42.2, 46.8) | -- | -- | -- |
| Ecuador 2012 | OW/OB | 16.4  (9.2, 23.6) | 38.1  (24.7, 51.5) | 13.2  (5.6, 20.8) | 16.5  (3.4, 29.6) | 18.2  (10.1, 26.3) | 1.5  (0, 4.5) | -- | -- |
|  | BAZ ≤ 2 | 25.3  (21.1, 29.5) | 43.7  (40.9, 46.4) | 12.7  (10.0, 15.3) | 15.4  (12.7, 18.0) | 27.0  (24.1, 29.8) | 0.5  (0, 1.1) | -- | -- |
| Mexico 2006 | OW/OB | 13.6  (6.3, 20.9) | 61.7  (51.8, 71.6) | 45.0  (33.1, 56.9) | -- | 41.1  (27.0, 55.2) | 6.9  (0.3, 13.4) | -- | -- |
|  | BAZ ≤ 2 | 21.2  (17.9, 24.5) | 48.8  (44.6, 53.0) | 34.3  (30.5, 38.1) | -- | 27.2  (22.9, 31.5) | 3.9  (2.3, 5.6) | -- | 2.8  (1.2, 4.4) |
| Mexico 2012 | OW/OB | 15.2  (7.8, 22.7) | 28.2  (19.9, 36.6) | 22.4  (15.5, 29.2) | 7.5  (1.7, 13.4) | -- | 2.3  (0, 6.7) | -- | 0.3  (0, 0.9) |
|  | BAZ ≤ 2 | 17.0  (14.8, 19.2) | 23.6  (20.4, 26.9) | 18.2  (15.2, 21.2) | 7.3  (5.5, 9.1) | -- | 0.2  (0, 0.4) | -- | 0.3  (0.0, 0.5) |
| Nicaragua 2005 | OW/OB | 20.1  (10.7, 29.5) | 24.6  (11.3, 37.8) | 59.4  (42.2, 76.7) | 1.2  (0, 2.9) | -- | -- | -- | -- |
|  | BAZ ≤ 2 | 20.3  (15.8, 24.8) | 22.8  (16.0, 29.6) | 44.2  (37.2, 51.1) | 0.8  (0.3, 1.2) | -- | -- | -- | -- |
| USA 2006 | OW/OB | 1.2  (0, 2.5) | 23.0  (15.53, 30.4) | 24.5  (15.7, 33.3) | -- | -- | 0.3  (0, 1.0) | 1.5  (0.1, 3.0) | -- |
|  | BAZ ≤ 2 | 2.0  (0.8, 3.2) | 13.4  (9.9, 16.9) | 11.8  (8.4, 15.2) | -- | -- | 0.5  (0.1, 0.9) | 0.8  (0.3, 1.2) | 0.2  (0, 0.4) |
| Afghanistan 2013 | OW/OB | 25.8  (10.5, 41.1) | 83.9  (64.8, 100) | 31.8  (11.6, 52.0) | 43.7  (34.8, 52.6) | 21.8  (13.7, 29.8) | -- | 58.9  (37.0, 80.9) | -- |
|  | BAZ ≤ 2 | 40.9  (34.0, 47.7) | 74.0  (68.7, 79.2) | 22.3  (17.0, 27.6) | 38.4  (32.7, 44.1) | 23.2  (17.5, 28.8) | -- | 42.2  (36.2, 48.2) | -- |
| Azerbaijan 2013 | OW/OB | 25.0  (16.6, 33.4) | 34.0  (25.3, 42.8) | 18.8  (11.2, 26.4) | -- | 16.8  (10.0, 23.5) | -- | -- | -- |
|  | BAZ ≤ 2 | 29.2  (24.9, 33.4) | 32.9  (28.8, 36.9) | 22.5  (18.4, 26.6) | -- | 8.1  (6.1, 10.2) | -- | -- | -- |
| Georgia 2009 | OW/OB | 25.7  (20.5, 31.0) | 0.6  (0, 1.7) | 0.6  (0, 1.7) | -- | -- | -- | -- | -- |
|  | BAZ ≤ 2 | 22.4  (18.9, 26.0) | 0.1  (0, 0.3) | 0.1  (0, 0.3) | -- | -- | -- | -- | -- |
| Pakistan 2011 | OW/OB | 64.2  (58.4, 69.9) | 90.4  (86.9, 93.9) | 53.3  (47.0, 59.5) | 59.9  (53.8, 66.1) | 49.7  (43.1, 56.3) | -- | 20.3  (15.3, 25.2) | -- |
|  | BAZ ≤ 2 | 63.1  (61.7, 64.5) | 89.2  (88.1, 90.2) | 51.5  (49.9, 53.1) | 51.4  (49.1, 53.6) | 49.6  (47.5, 51.8) | -- | 18.2  (16.6, 19.7) | -- |

^1^Values represent % (95% confidence interval). Surveys are listed in alphabetical order within geographic categories, consistent with Table 3. BAZ, BMI-for-age Z-score; MDI, micronutrient deficiency index; OW/OB, overweight or obese, defined as BMI-for-age Z-score > 2 SD; PNG, Papua New Guinea.

**Supplementary Table 4. Unadjusted (bivariate) odds ratios for the intra-individual double burden of malnutrition, defined as concomitant overweight/obesity and micronutrient deficiency index (MDI) > 0, according to individual and household characteristics, by survey^1^**

| Country, Survey year | Age ≥ 24.0 months (reference =  6-23.9 mo) | Male sex  (reference =  female) | Urban  (reference =  rural) | Medium SES  (reference =  low SES) | High SES (reference =  low SES) | High caregiver education (reference =  low education) |
| --- | --- | --- | --- | --- | --- | --- |
| Bangladesh 2010 | -- | -- | -- | -- | -- | -- |
| Bangladesh 2012 | -- | -- | -- | -- | -- | -- |
| Cambodia 2014 | -- | -- | -- | -- | -- | -- |
| Laos 2006 | -- | -- | -- | -- | -- | -- |
| PNG 2005 | -- | -- | -- | -- | -- | -- |
| Philippines 2011 | -- | 1.43 (0.29, 7.21) | 1.11 (0.30, 4.12) | 1.48 (0.18, 2.25) | -- | 1.99 (0.4, 9.62) |
| Vietnam 2010 | -- | -- | -- | -- | -- | -- |
| Cote d'Ivoire 2007 | 0.28 (0.10, 0.84)* | 2.37 (0.75, 7.54) | 1.30 (0.50, 3.39) | 1.36 (0.47, 3.94) | 0.27 (0.03, 2.29) | 0.31 (0.04, 2.33) |
| Cameroon 2009 | 1.61 (0.60, 4.31) | 4.79 (1.59, 14.44)** | 1.02 (0.29, 3.65) | 1.09 (0.42, 2.86) | 0.99 (0.28, 3.51) | 1.26 (0.50, 3.19) |
| Kenya 2007 | 0.96 (0.45, 2.02) | 1.14 (0.53, 2.46) | -- | 0.63 (0.24, 1.66) | 1.18 (0.49, 2.84) | 1.21 (0.44, 3.35) |
| Kenya 2010 | 0.72 (0.29, 1.82) | 0.74 (0.31, 1.77) | -- | 0.25 (0.07, 0.96)* | 1.48 (0.55, 3.97) | 0.89 (0.24, 3.28) |
| Liberia 2011 | 3.21 (1.03, 10.05)* | 0.84 (0.35, 1.99) | 2.66 (0.7, 9.87) | 1.57 (0.31, 8.23) | 3.32 (0.95, 11.58) | -- |
| Malawi 2016 | 0.28 (0.12, 0.66) | 3.66 (1.30, 10.34) | 6.00 (2.49, 14.45)** | 0.49 (0.59, 3.77) | 6.27 (2.90, 17.95)** | 2.86 (1.44, 5.67)** |
| Colombia 2010 | 0.34 (0.20, 0.58)** | 1.44 (0.85, 2.42) | 0.72 (0.43, 1.22) | 0.69 (0.39, 1.21) | 0.31 (0.09, 1.10) | 1.21 (0.63, 2.35) |
| Ecuador 2012 | 1.26 (0.63, 2.53) | 1.49 (0.70, 3.16) | 0.75 (0.31, 1.80) | 0.69 (0.18, 2.58) | 1.09 (0.34, 3.52) | 1.14 (0.48, 2.74) |
| Mexico 2006 | 1.06 (0.46, 2.46) | 1.88 (0.97, 3.64) | 0.62 (0.31, 1.23) | 0.47 (0.22, 0.996)* | 0.61 (0.20, 1.91) | 0.52 (0.25, 1.07) |
| Mexico 2012 | 1.05 (0.45, 2.43) | 1.35 (0.66, 2.74) | 1.14 (0.59, 2.18) | 1.22 (0.59, 2.50) | 0.98 (0.24, 3.93) | -- |
| Nicaragua 2005 | 0.84 (0.43, 1.65) | 1.05 (0.44, 2.51) | 1.56 (0.61, 4.00) | -- | -- | 2.39 (1.03, 5.54)* |
| USA 2006 | 0.64 (0.26, 1.58) | 2.26 (0.94, 5.40) | -- | 0.41 (0.12, 1.40) | 1.08 (0.31, 3.80) | 0.50 (0.16, 1.53) |
| Afghanistan 2013 | 1.21 (0.67, 2.21) | 0.32 (0.16, 0.64)** | -- | 0.68 (0.18, 2.61) | 0.55 (0.16, 1.87) | -- |
| Azerbaijan 2013 | 0.55 (0.30, 1.01) | 1.82 (0.96, 3.45) | 1.24 (0.64, 2.39) | 0.86 (0.41, 1.79) | 0.40 (0.14, 1.11) | -- |
| Georgia 2009 | -- | -- | -- | -- | -- | -- |
| Mongolia 2006 | 0.51 (0.14, 1.86) | 0.63 (0.21, 1.92) | 0.76 (0.25, 2.34) | -- | -- | 0.57 (0.16, 2.02) |
| Pakistan 2011 | 0.76 (0.58, 0.99)* | 1.19 (0.93, 1.55) | 0.86 (0.63, 1.17) | 0.72 (0.54, 0.96)* | 1.01 (0.69, 1.48) | 0.75 (0.52, 1.10) |

^1^Values represent odds ratio (95% confidence interval). Surveys are listed in alphabetical order within geographic categories, consistent with Figure 2. Surveys for which the number of cases of double burden of malnutrition was < 10 were excluded (Bangladesh 2010; Bangladesh 2012; Cambodia; Georgia; Laos; Papua New Guinea; Vietnam). Missing cells indicate that the variable was unavailable in the dataset, or that one level of the variable had 0 observations. * indicates P < 0.05; ** indicates P < 0.01

^2^Educational level of the head of household used because maternal education was not available in Colombia, Mexico 2006, and the United States.

^3^SES was recoded for the Philippines to be a 2-level categorical variable combining middle and high SES categories. SES, socioeconomic status.

**Supplementary Table 5. Unadjusted (bivariate) odds ratios for the intra-individual double burden of malnutrition, defined as concomitant overweight/obesity and anemia, according to individual and household characteristics, by survey^1^**

| Country, Survey year | Age ≥ 24.0 months (reference =  6-23.9 mo) | Male sex  (reference =  female) | Urban  (reference =  rural) | Medium SES  (reference =  low SES) | High SES (reference =  low SES) | High caregiver education  (reference =  low education) |
| --- | --- | --- | --- | --- | --- | --- |
| Bangladesh 2010 | -- | 1.17 (0.43, 3.18) |  |  |  |  |
| Bangladesh 2012 | -- | -- | -- | -- | -- | -- |
| Cambodia 2014 | -- | -- | -- | -- | -- | -- |
| Laos 2006 | -- | -- | -- | -- | -- | -- |
| PNG 2005 | 4.40 (0.52, 37.39) | 1.20 (0.38, 3.75) | <0.001 | 2.01 (0.49, 8.20) | 1.55 (0.22, 10.89) |  |
| Philippines 2011 | -- | 1.44 (0.25, 8.45) | 1.91 (0.40, 9.08) | 0.06 (0.01, 0.50)* | -- | 0.91 (0.14, 5.89) |
| Vietnam 2010 | -- | -- | -- | -- | -- | -- |
| Cote d'Ivoire 2007 | 0.40 (0.15, 0.77) | 0.84 (0.43, 1.65) | 1.19 (0.54, 2.62) | 1.08 (0.47, 2.47) | 0.55 (0.16, 1.90) | 0.50 (0.12, 2.16) |
| Cameroon 2009 | 1.25 (0.29, 5.42) | 17.11 (2.10, 139.24)** | 1.52 (0.40, 5.78) | 0.64 (0.14, 2.97) | 0.27 (0.03, 2.26) | 0.82 (0.22, 2.98) |
| Kenya 2007 | 1.17 (0.54, 2.54) | 1.18 (0.51, 2.76) | -- | 0.60 (0.21, 1.72) | 0.97 (0.41, 2.32) | 0.77 (0.25, 2.36) |
| Kenya 2010 | 1.03 (0.48, 2.18) | 1.33 (0.67, 2.67) | -- | 0.28 (0.10, 0.81)* | 1.00 (0.40, 2.55) | 0.93 (0.32, 2.67) |
| Liberia 2011 | 5.34 (1.83, 15.60)** | 0.63 (0.22, 1.82) | 1.44 (0.55, 3.77) | 1.68 (0.41, 6.90) | 1.57 (0.39, 6.38) | -- |
| Malawi 2016 | 0.25 (0.08, 0.75)* | 3.29 (1.07, 10.15)* | 4.75 (2.41, 9.37)** | 1.62 (0.57, 4.63) | 3.95 (0.75, 20.86) | 3.69 (1.52, 8.96)* |
| Colombia 2010 | 0.13 (0.05, 0.36)** | 2.23 (0.80, 6.22) | 0.47 (0.17, 1.28) | 0.43 (0.10, 1.87) | 0.89 (0.18, 4.46) | 1.40 (0.34, 5.85) |
| Ecuador 2012 | 0.57 (0.19, 1.74) | 0.89 (0.35, 2.24) | 0.59 (0.22, 1.62) | 0.13 (0.03, 0.61)** | 0.24 (0.05, 1.08) | 0.51 (0.19, 1.39) |
| Mexico 2006 | 0.99 (0.23, 3.94) | 1.38 (0.44, 4.32) | 0.33 (0.10, 1.10) | 0.44 (0.09, 2.04) | 1.04 (0.3, 8.50) | 0.70 (0.22, 2.23) |
| Mexico 2012 | 0.33 (0.14, 0.77)* | 1.75 (0.58, 5.33) | 2.57 (0.92, 7.19) | 0.91 (0.31, 2.67) | 3.18 (0.73, 13.89) |  |
| Nicaragua 2005 | 0.50 (0.12, 2.17) | 0.77 (0.17, 3.40) | 0.53 (0.16, 1.75) |  |  | 0.36 (0.10, 1.24) |
| USA 2006 | -- | -- | -- | -- | -- | -- |
| Afghanistan 2013 | 0.66 (0.23, 1.91) | 0.29 (0.11, 0.79)* |  | 0.34 (0.10, 1.11) | 0.22 (0.06, 0.83)* |  |
| Azerbaijan 2013 | 0.34 (0.16, 0.74) | 0.95 (0.44, 2.05) | 0.72 (0.33, 1.59) | 0.38 (0.17, 0.86)* | 0.24 (0.07, 0.77)* |  |
| Georgia 2009 | 0.23 (0.15, 0.37)** | 1.12 (0.75, 1.68) | 0.79 (0.47, 1.31) | -- | -- | -- |
| Mongolia 2006 | -- | -- | -- | -- | -- | -- |
| Pakistan 2011 | 0.73 (0.54, 1.00)* | 1.27 (0.94, 1.73) | 0.79 (0.55, 1.13) | 0.66 (0.46, 0.94)* | 0.91 (0.61, 1.37) | 0.74 (0.49, 1.14) |

^1^Values represent odds ratio (95% confidence interval). Surveys are listed in alphabetical order within geographic categories, consistent with Figure 2. Surveys for which the number of cases of double burden of malnutrition was < 10 were excluded (Bangladesh 2012; Cambodia; Laos; Mongolia; United States; Vietnam). Missing cells indicate that the variable was unavailable in the dataset, or that one level of the variable had 0 observations. * indicates P < 0.05; ** indicates P < 0.01

^2^Educational level of the head of household used because maternal education was not available in Colombia and Mexico 2006

^3^SES was recoded for the Philippines to be a 2-level categorical variable combining middle and high SES categories. SES, socioeconomic status.

**Supplementary Table 6. Adjusted odds ratios for predictors of the intra-individual double burden of malnutrition, defined as concomitant overweight/obesity and MDI > 0, according to individual and household characteristics, by survey^1^**

| Country, Survey year | Age ≥ 24.0 months (reference =  6-23.9 mo) | Male sex  (reference =  female) | Urban  (reference =  rural) | Medium SES  (reference =  low SES) | High SES (reference =  low SES) | High caregiver education  (reference =  low education) |
| --- | --- | --- | --- | --- | --- | --- |
| Bangladesh 2010 | -- | 2.60 (0.50, 13.42) | -- | -- | -- | -- |
| Bangladesh 2012 | 0.24 (0.01, 4.13) | 4.14 (0.62, 27.60) | 2.89 (0.31, 27.07) | 0.97 (0.15, 6.31) | 2.47 (1.00, 6.31) | 0.34 (0.08, 1.54) |
| Cambodia 2014 | -- | -- | -- | -- | -- | -- |
| Laos 2006 | -- | -- | -- | -- | -- | -- |
| PNG 2005 | 0.73 (0.07, 8.09) | 2.40 (0.22, 25.54) | -- | -- | -- | -- |
| Philippines 2011 | -- | 1.44 (0.33, 6.38) | 1.09 (0.29, 4.04) | 1.39 (0.19, 9.96) | | 1.89 (0.40, 8.95) |
| Vietnam 2010 | 0.29 (0.05, 1.57) | 1.00 (0.25, 4.00) | 2.48 (0.43, 14.39) | -- | -- | -- |
| Cote d'Ivoire 2007 | 0.31 (0.11, 0.89)* | 2.22 (0.72, 6.79) | 1.98 (0.61, 6.40) | 0.98 (0.25, 3.84) | 0.21 (0.02, 2.46) | 0.44 (0.04, 4.43) |
| Cameroon 2009 | 1.66 (0.58, 4.73) | 4.83 (1.62, 14.39)** | 0.98 (0.19, 4.99) | 1.02 (0.32,3.24) | 0.86 (0.16, 4.63) | 1.37 (0.39, 4.88) |
| Kenya 2007 | 0.98 (0.45, 2.11) | 1.16 (0.54, 2.49) | -- | 0.62 (0.23, 1.65) | 1.14 (0.46, 2.84) | 1.19 (0.42, 3.41) |
| Kenya 2010 | 0.75 (0.29, 1.93) | 0.75 (0.30, 1.90) | -- | 0.25 (0.07, 0.97)* | 1.57 (0.58, 4.25) | 0.76 (0.21, 2.72) |
| Liberia 2011 | 3.32 (1.07, 10.31)* | 0.87 (0.36, 2.09) | 2.00 (0.39, 10.15) | 1.19 (0.30, 4.67) | 1.95 (0.46, 8.27) | -- |
| Malawi 2016 | 0.40 (0.19, 0.86)* | 3.38 (1.08, 10.51)* | 3.04 (0.64, 14.50) | 1.53 (0.57, 4.14) | 2.61 (0.71, 9.58) | 1.29 (0.58, 2.85) |
| Colombia 2010 | 0.61 (0.28, 1.34) | 1.37 (0.70, 2.70) | 0.65 (0.26, 1.62) | 1.26 (0.48, 3.27) | 1.33 (0.24, 7.26) | 1.20 (0.61, 2.33) |
| Ecuador 2012 | 1.26 (0.63, 2.53) | 1.44 (0.70, 2.97) | 0.77 (0.27, 2.23) | 0.71 (0.20, 2.49) | 1.11 (0.27, 4.62) | 1.27 (0.59, 2.72) |
| Mexico 2006 | 1.03 (0.44, 2.40) | 1.91 (0.99, 3.69) | 0.79 (0.34, 1.83) | 0.55 (0.24, 1.27) | 0.84 (0.24, 3.01) | 0.63 (0.29, 1.36) |
| Mexico 2012 | 1.05 (0.45, 2.47) | 1.35 (0.67, 2.73) | 1.11 (0.55, 2.27) | 1.18 (0.55, 2.54) | 0.93 (0.23, 3.87) | -- |
| Nicaragua 2005 | 0.89 (0.44, 1.80) | 1.06 (0.44, 2.55) | 1.09 (0.45, 2.66) | -- | -- | 2.29 (1.04, 5.03)* |
| USA 2006 | 0.46 (0.19, 1.10) | 1.66 (0.65, 4.26) | -- | 0.45 (0.13, 1.58) | 1.27 (0.34, 4.81) | -- |
| Afghanistan 2013 | 1.21 (0.58, 2.53) | 0.36 (0.17, 0.75)** | -- | 0.67 (0.17, 2.60) | 0.55 (0.15, 1.94) | -- |
| Azerbaijan 2013 | 0.51 (0.27, 0.97)* | 2.00 (1.04, 3.83)* | 1.72 (0.82, 3.61) | 0.74 (0.34, 1.61) | 0.29 (0.10, 0.88)* | -- |
| Georgia 2009 | -- | -- | -- | -- | -- | -- |
| Mongolia 2006 | 0.55 (0.15, 2.07) | 0.68 (0.22, 2.15) | 0.83 (0.26, 2.62) | -- | -- | 0.61 (0.17, 2.23) |
| Pakistan 2011 | 0.74 (0.57, 0.97)* | 1.19 (0.92, 1.55) | 0.91 (0.63, 1.29) | 0.76 (0.56, 1.05) | 1.34 (0.81, 2.23) | 0.67 (0.43, 1.06) |

^1^Values represent odds ratio (95% confidence interval); each value is adjusted for all other characteristics shown. Surveys for which the number of cases of double burden of malnutrition was < 10 were excluded (Bangladesh 2010; Bangladesh 2012; Cambodia; Georgia; Laos; Papua New Guinea; Vietnam). Missing cells indicate that the variable was unavailable in the dataset, or that one level of the variable had 0 observations. * indicates P < 0.05; ** indicates P < 0.01

^2^Educational level of the head of household used because maternal education was not available in Colombia, Mexico 2006, and the United States

^3^SES was recoded for the Philippines to be a 2-level categorical variable combining middle and high SES categories. SES, socioeconomic status.

**Supplementary Table 7. Adjusted odds ratios for predictors of the intra-individual double burden of malnutrition, defined as concomitant overweight/obesity and anemia, according to individual and household characteristics, by survey^1^**

| Country, Survey year | Age ≥ 24.0 months (reference =  6-23.9 mo) | Male sex  (reference =  female) | Urban  (reference =  rural) | Medium SES  (reference =  low SES) | High SES (reference =  low SES) | High caregiver education  (reference =  low education) |
| --- | --- | --- | --- | --- | --- | --- |
| Bangladesh 2010 | -- | 1.17 (0.43, 3.17) | -- | -- | -- | -- |
| Bangladesh 2012 | 22.81 (1.69, 307.48)* | -- | 1.33 (0.13, 13.47) | 1.30 (0.12, 13.73) | 0.12 (0.01, 1.49) | 0.03 (0.00, 0.31)** |
| Cambodia 2014 | -- | -- | -- | -- | -- | -- |
| Laos 2006 | -- | -- | -- | -- | -- | -- |
| PNG 2005 | 4.37 (0.52, 37.01) | 1.19 (0.37, 3.80) | -- | 1.99 (0.49, 8.15) | 1.46 (0.21, 10.07) | -- |
| Philippines 2011 | -- | 1.34 (0.25, 7.18) | 1.90 (0.40, 9.06) | 0.06 (0.01, 0.51)* | | 1.03 (0.17, 6.09) |
| Vietnam 2010 | -- | -- | -- | -- | -- | -- |
| Cote d'Ivoire 2007 | 0.35 (0.16, 0.78)* | 0.79 (0.42, 1.47) | 1.80 (0.76, 4.24) | 0.81 (0.32, 2.01) | 0.41 (0.10, 1.69) | 0.58 (0.12, 2.78) |
| Cameroon 2009 | 1.26 (0.30, 5.33) | 17.47 (2.08, 146.70)** | 2.53 (0.61, 10.53) | 0.44 (0.11, 1.78) | 0.16 (0.02, 1.55) | 1.10 (0.24, 4.98) |
| Kenya 2007 | 1.21 (0.55, 2.67) | 1.20 (0.52, 2.81) | -- | 0.60 (0.21, 1.75) | 0.99 (0.39, 2.51) | 0.77 (0.23, 2.54) |
| Kenya 2010 | 0.95 (0.42, 2.15) | 1.26 (0.61, 2.63) | -- | 0.23 (0.07, 0.72)* | 1.03 (0.40, 2.63) | 0.87 (0.31, 2.47) |
| Liberia 2011 | 5.37 (1.85, 15.55)** | 0.66 (0.22, 1.92) | 1.30 (0.45, 3.70) | 1.55 (0.32, 7.51) | 1.33 (0.28, 6.44) | -- |
| Malawi 2016 | 0.34 (0.14, 0.86)* | 3.20 (0.95, 10.79) | 2.34 (0.68, 8.08) | 1.44 (0.54, 3.83) | 1.27 (0.16, 10.08) | 2.31 (1.002, 5.32)* |
| Colombia 2010 | 0.14 (0.03, 0.67)* | 2.92 (0.56, 15.13) | 0.33 (0.06, 1.89) | 1.06 (0.08, 13.81) | 6.14 (0.47, 80.98) | 1.30 (0.31, 5.29) |
| Ecuador 2012 | 0.58 (0.18, 1.83) | 0.90 (0.34, 2.13) | 1.01 (0.39, 2.64) | 0.14 (0.02, 0.84)* | 0.26 (0.05, 1.49) | 0.78 (0.25, 2.47) |
| Mexico 2006 | 0.92 (0.21, 4.05) | 1.41 (0.44, 4.51) | 0.31 (0.12, 0.81)* | 0.64 (0.18, 2.22) | 2.37 (0.31, 18.65) | 0.95 (0.39, 2.32) |
| Mexico 2012 | 0.33 (0.14, 0.80)* | 1.75 (0.57, 5.38) | 2.23 (0.71, 7.00) | 0.76 (0.24, 2.46) | 2.50 (0.52, 12.09) | -- |
| Nicaragua 2005 | 0.47 (0.10, 2.17) | 0.76 (0.17, 3.43) | 0.72 (0.20, 2.58) | -- | -- | 0.40 (0.11, 1.43) |
| USA 2006 | 0.93 (0.07, 11.97) | 0.68 (0.05, 8.60) | -- | -- | -- | -- |
| Afghanistan 2013 | 0.75 (0.26, 2.18) | 0.30 (0.11, 0.83)* | -- | 0.35 (0.11, 1.15) | 0.22 (0.06, 0.85)* | -- |
| Azerbaijan 2013 | 0.31 (0.14, 0.70)** | 1.02 (0.48, 2.16) | 1.13 (0.48, 2.63) | 0.35 (0.15, 0.84)* | 0.21 (0.06, 0.72)* | -- |
| Georgia 2009 | 0.23 (0.15, 0.36)** | 1.11 (0.74, 1.67) | 0.74 (0.44, 1.23) | -- | -- | -- |
| Mongolia 2006 | 2.32 (0.06, 84.13) | 0.81 (0.02, 28.01) | -- | -- | -- | 0.20 (0.01, 7.36) |
| Pakistan 2011 | 0.72 (0.53, 0.98)* | 1.27 (0.93, 1.72) | 0.86 (0.56, 1.31) | 0.70 (0.48, 1.02) | 1.21 (0.70, 2.09) | 0.71 (0.42, 1.20) |

^1^Values represent odds ratio (95% confidence interval); each value is adjusted for all other characteristics shown. Surveys are listed in alphabetical order within geographic categories, consistent with Figure 2. Surveys for which the number of cases of double burden of malnutrition was < 10 were excluded (Bangladesh 2012; Cambodia; Laos; Mongolia; United States; Vietnam). Missing cells indicate that the variable was unavailable in the dataset, or that one level of the variable had 0 observations. * indicates P < 0.05; ** indicates P < 0.01

^2^Educational level of the head of household used because maternal education was not available in Colombia and Mexico 2006

^3^SES was recoded for the Philippines to be a 2-level categorical variable combining middle and high SES categories. SES, socioeconomic status.

**Supplementary Table 8. Adjusted odds ratios for predictors of micronutrient deficiency index > 0 (i.e., presence of at least one micronutrient deficiency), according to individual and household characteristics, by survey^1^**

| Country, Survey year | Age ≥ 24.0 months (reference =  6-23.9 mo) | Male sex  (reference =  female) | Urban  (reference =  rural) | Medium SES  (reference =  low SES) | High SES (reference =  low SES) | High caregiver education  (reference =  low education) |
| --- | --- | --- | --- | --- | --- | --- |
| Bangladesh 2010 | -- | 1.74 (1.32, 2.31)** | -- | -- | -- | -- |
| Bangladesh 2012 | 0.64 (0.23, 1.80) | 0.81 (0.49, 1.33) | 0.90 (0.43, 1.87) | 0.82 (0.37, 1.81) | 0.51 (0.15, 1.76) | 1.25 (0.52, 2.96) |
| Cambodia 2014 | 1.12 (0.62, 2.02) | 1.02 (0.58, 1.82) | 0.57 (0.28, 1.15) | 1.17 (0.67, 2.05) | 3.28 (1.19, 9.00)* | 0.84 (0.46, 1.51) |
| Laos 2006 | 0.13 (0.07, 0.23)** | 1.67 (0.89, 3.15) | 0.68 (0.24, 1.91) | 1.07 (0.49, 2.36) | 1.22 (0.29, 5.10) | 0.48 (0.18, 1.31) |
| PNG 2005 | 0.79 (0.52, 1.21) | 1.29 (0.85, 1.95) | 0.96 (0.49, 1.87) | 0.87 (0.43, 1.77) | 0.79 (0.41, 1.53) | -- |
| Philippines 2011 | -- | 1.17 (0.85, 1.61) | 0.75 (0.55, 1.02) | 0.39 (0.26, 0.58)** | | 0.54 (0.40, 0.73)** |
| Vietnam 2010 | 0.51 (0.29, 0.90)* | 0.89 (0.59, 1.33) | 0.75(0.46, 1.23) | -- | -- | -- |
| Cote d'Ivoire 2007 | 0.38 (0.27, 0.53)** | 1.18 (0.79, 1.75) | 2.29 (1.29, 4.06)** | 0.84 (0.50, 1.41) | 0.60 (0.28, 1.27) | 1.04 (0.64, 1.67) |
| Cameroon 2009 | 0.92 (0.55, 1.56) | 1.16 (0.81, 1.66) | 0.54 (0.32, 0.91)* | 0.77 (0.49, 1.21) | 0.89 (0.42, 1.87) | 0.57 (0.34, 0.95)* |
| Kenya 2007 | 0.76 (0.56, 1.04) | 1.55 (1.11, 2.17)* | -- | 1.18 (0.82, 1.71) | 1.49 (1.01, 2.21)* | 1.17 (0.68, 2.01) |
| Kenya 2010 | 0.71 (0.53, 0.96)* | 1.41 (1.09, 1.83)* | -- | 0.71 (0.52, 0.98)* | 0.85 (0.56, 1.29) | 0.83 (0.59, 1.15) |
| Liberia 2011 | 0.62 (0.47, 0.81)** | 1.02 (0.82, 1.26) | 1.26 (0.86, 1.83) | 1.24 (0.83, 1.8) | 1.12 (0.72, 1.73) | -- |
| Malawi 2016 | 0.53 (0.36, 0.76)** | 1.40 (0.97, 2.01) | 3.38 (1.58, 7.22)** | 0.95 (0.65, 1.41) | 0.76 (0.37, 1.55) | 0.79 (0.40, 1.54) |
| Colombia 2010 | 0.53 (0.38, 0.73)** | 1.44 (1.14, 1.83)** | 0.86 (0.63, 1.18) | 0.69 (0.50, 0.94)* | 0.49 (0.28, 0.87)* | 0.85 (0.67, 1.08) |
| Ecuador 2012 | 0.52 (0.40, 0.69)** | 1.19 (0.94, 1.52) | 1.33 (1.02, 1.73)* | 0.92 (0.63, 1.33) | 0.56 (0.39, 0.82)** | 0.94 (0.67, 1.31) |
| Mexico 2006 | 0.68 (0.45, 1.02) | 1.34 (0.99, 1.81) | 1.03 (0.74, 1.44) | 0.85 (0.60, 1.24) | 0.47 (0.21, 1.08) | 0.88 (0.65, 1.21) |
| Mexico 2012 | 0.45 (0.32, 0.62)** | 1.12 (0.86, 1.45) | 1.05 (0.78, 1.40) | 0.77 (0.57, 1.05) | 0.89 (0.55, 1.43) | -- |
| Nicaragua 2005 | 0.52 (0.38, 0.71)** | 0.97 (0.67, 1.41) | 1.44 (0.72, 2.89) | -- | -- | 1.60 (1.07, 2.41)* |
| USA 2006 | 0.40 (0.27, 0.61)** | 1.17 (0.78, 1.73) | -- | 0.77 (0.50, 1.17) | 0.66 (0.30, 1.42) | 0.59 (0.32, 1.09) |
| Afghanistan 2013 | 1.23 (0.82, 1.86) | 1.25 (0.64, 2.45) | -- | 0.96 (0.42, 2.16) | 0.45 (0.25, 0.82)* | -- |
| Azerbaijan 2013 | 0.56 (0.39, 0.82)** | 1.38 (0.97, 1.97) | 1.54 (1.08, 2.20)* | 0.79 (0.53, 1.17) | 0.63 (0.40, 0.99)* | -- |
| Georgia 2009 | -- | -- | -- | -- | -- | -- |
| Mongolia 2006 | 0.14 (0.03, 0.67)* | 1.38 (0.35, 5.51) | 0.68 (0.17, 2.68) | -- | -- | 3.67 (0.77, 17.45) |
| Pakistan 2011 | 1.09 (0.91, 1.30) | 0.94 (0.78, 1.12) | 1.00 (0.81, 1.25) | 1.07 (0.86, 1.33) | 1.00 (0.75, 1.34) | 0.81 (0.63, 1.03) |

^1^Values represent odds ratio (95% confidence interval); each value is adjusted for all other characteristics shown. Surveys are listed in alphabetical order within geographic categories, consistent with Figure 2. Surveys for which the number of cases of MDI > 0 was < 10 were excluded (Georgia). Missing cells indicate that the variable was unavailable in the dataset, or that one level of the variable had 0 observations. * indicates P < 0.05; ** indicates P < 0.01

^2^Educational level of the head of household used because maternal education was not available in Colombia, Mexico 2006, and the United States

^3^SES was recoded for the Philippines to be a 2-level categorical variable combining middle and high SES categories. SES, socioeconomic status.

**Supplementary Table 9. Adjusted odds ratios for predictors of anemia, according to individual and household characteristics, by survey^1^**

| Country, Survey year | Age ≥ 24.0 months (reference =  6-23.9 mo) | Male sex  (reference =  female) | Urban  (reference =  rural) | Medium SES  (reference =  low SES) | High SES (reference =  low SES) | High caregiver education  (reference =  low education) |
| --- | --- | --- | --- | --- | --- | --- |
| Bangladesh 2010 | -- | 1.29 (1.10, 1.52)** | -- | -- | -- | -- |
| Bangladesh 2012 | 0.87 (0.32, 2.36) | 1.07 (0.62, 1.86) | 0.35 (0.15, 0.83)* | 1.73 (0.74, 4.04) | 2.54 (0.63, 10.26) | 0.61 (0.24, 1.56) |
| Cambodia 2014 | 0.32 (0.13, 0.78)* | 0.73 (0.48, 1.09) | 1.85 (0.54, 6.36) | 0.63 (0.34, 1.20) | 0.35 (0.10, 1.17) | 1.00 (0.58, 1.74) |
| Laos 2006 | 0.33 (0.22, 0.48)** | 1.37 (0.89, 2.11) | 0.30 (0.09, 1.02) | 1.13 (0.56, 2.30) | 1.46 (0.34, 6.27) | 1.06 (0.55, 2.05) |
| PNG 2005 | 0.43 (0.32, 0.58)** | 1.04 (0.79, 1.39) | 0.73 (0.33, 1.57) | 1.28 (0.61, 2.71) | 0.95 (0.48, 1.89) | -- |
| Philippines 2011 | -- | 0.97 (0.72, 1.30) | 1.06 (0.84, 1.34) | 0.73 (0.49, 1.08) | | 0.48 (0.36, 0.65)** |
| Vietnam 2010 | 0.26 (0.13, 0.54)** | 1.30 (0.58, 2.95) | 0.89 (0.35, 2.26) | -- | -- | -- |
| Cote d'Ivoire 2007 | 0.38 (0.24, 0.61)** | 0.99 (0.71, 1.37) | 0.91 (0.42, 1.97) | 0.72 (0.44, 1.16) | 0.55 (0.24, 1.26) | 1.23 (0.71, 2.13) |
| Cameroon 2009 | 0.50 (0.37, 0.69)** | 1.34 (0.98, 1.84) | 1.06 (0.60, 1.88) | 0.49 (0.27, 0.89)* | 0.36 (0.17, 0.76)** | 0.51 (0.33, 0.77)** |
| Kenya 2007 | 0.66 (0.49, 0.89)** | 1.22 (0.88, 1.68) | -- | 1.43 (1.06, 1.93)* | 1.24 (0.89, 1.74) | 0.53 (0.35, 0.81)** |
| Kenya 2010 | 0.66 (0.49, 0.91)* | 1.51 (1.05, 2.15)* | -- | 0.82 (0.53, 1.26) | 0.57 (0.35, 0.93)* | 1.06 (0.68, 1.64) |
| Liberia 2011 | 0.90 (0.71, 1.14) | 1.26 (1.00, 1.58) | 0.77 (0.55, 1.09) | 1.35 (0.93, 1.95) | 1.01 (0.67, 1.53) | -- |
| Malawi 2016 | 0.34 (0.20, 0.58)** | 1.12 (0.73, 1.71) | 1.37 (0.83, 2.27) | 0.88 (0.56, 1.40) | 0.55 (0.21, 1.45) | 1.21 (0.73, 1.98) |
| Colombia 2010 | 0.47 (0.32, 0.69)** | 1.16 (0.83, 1.62) | 1.21 (0.77, 1.88) | 0.64 (0.40, 1.05) | 0.41 (0.15, 1.17) | 1.24 (0.88, 1.74) |
| Ecuador 2012 | 0.16 (0.12, 0.23)** | 1.32 (0.97, 1.79) | 1.84 (1.23, 2.76)** | 0.50 (0.37, 0.68)** | 0.11 (0.05, 0.26)** | 0.88 (0.60, 1.30) |
| Mexico 2006 | 0.45 (0.30, 0.70)** | 0.80 (0.54, 1.19) | 0.93 (0.64, 1.36) | 0.55 (0.34, 0.91)* | 0.28 (0.10, 0.81)* | 0.99 (0.66, 1.48) |
| Mexico 2012 | 0.48 (0.33, 0.70)** | 1.32 (0.94, 1.86) | 1.17 (0.86, 1.61) | 0.68 (0.49, 0.94)* | 0.76 (0.42, 1.40) | -- |
| Nicaragua 2005 | 0.33 (0.22, 0.51)** | 1.23 (0.84, 1.81) | 0.65 (0.38, 1.10) | -- | -- | 0.58 (0.38, 0.80)* |
| USA 2006 | 0.53 (0.26, 1.08) | 0.47 (0.18, 1.18) | -- | 0.48 (0.20, 1.16) | 0.44 (0.06, 3.36) | 0.43 (0.13, 1.43) |
| Afghanistan 2013 | 1.06 (0.69, 1.63) | 0.62 (0.41, 0.93)* | -- | 0.73 (0.33, 1.63) | 0.61 (0.32, 1.17) | -- |
| Azerbaijan 2013 | 0.51 (0.35, 0.75)** | 1.61 (1.12, 2.31)* | 0.84 (0.57, 1.26) | 1.00 (0.62, 1.62) | 0.67 (0.37, 1.21) | -- |
| Georgia 2009 | 0.39 (0.31, 0.50)** | 1.03 (0.80, 1.33) | 0.95 (0.64, 1.42) | -- | -- | -- |
| Mongolia 2006 | 0.27 (0.10, 0.73)** | 1.40 (0.61, 3.21) | 0.39 (0.17, 0.92)* | -- | -- | 0.86 (0.32, 2.31) |
| Pakistan 2011 | 0.55 (0.49, 0.62)** | 1.05 (0.94, 1.17) | 1.38 (1.19, 1.59)** | 0.71 (0.62, 0.82)** | 0.60 (0.50, 0.73)** | 0.88 (0.75, 1.04) |

^1^Values represent odds ratio (95% confidence interval); each value is adjusted for all other characteristics shown. Surveys are listed in alphabetical order within geographic categories, consistent with Figure 2. Missing cells indicate that the variable was unavailable in the dataset, or that one level of the variable had 0 observations. * indicates P < 0.05; ** indicates P < 0.01

^2^Educational level of the head of household used because maternal education was not available in Colombia, Mexico 2006, and the United States

^3^SES was recoded for the Philippines to be a 2-level categorical variable combining middle and high SES categories. SES, socioeconomic status.

**Supplementary Table 10. Adjusted odds ratios for predictors of overweight and obesity (BMI-for-age Z-score > 2 SD), according to individual and household characteristics, by survey^1^**

| Country, Survey year | Age ≥ 24.0 months (reference =  6-23.9 mo) | Male sex  (reference =  female) | Urban  (reference =  rural) | Medium SES  (reference =  low SES) | High SES (reference =  low SES) | High caregiver education  (reference =  low education) |
| --- | --- | --- | --- | --- | --- | --- |
| Bangladesh 2010 | -- | 1.59 (0.95, 2.64) | -- | -- | -- | -- |
| Bangladesh 2012 | 0.74 (0.22, 2.46) | 1.49 (0.44, 5.11) | 1.29 (0.44, 3.81) | 3.30 (0.97, 11.28) | 5.59 (1.86, 16.82) | 0.61 (0.22, 1.66) |
| Cambodia 2014 | -- | -- | -- | -- | -- | -- |
| Laos 2006 | -- | -- | -- | -- | -- | -- |
| PNG 2005 | 1.97 (1.27, 3.04)** | 1.89 (1.26, 2.84)** | 1.19 (0.47, 3.00) | 0.66 (0.31, 1.37) | 1.14 (0.58, 2.23) | -- |
| Philippines 2011 | -- | 1.05 (0.69, 1.60) | 1.43 (0.89, 2.31) | 1.37 (0.88, 2.13) | | 1.97 (1.21, 3.19)** |
| Vietnam 2010 | 0.42 (0.19, 0.93)* | 1.18 (0.65, 2.17) | 2.24 (1.01, 4.93) | -- | -- | -- |
| Cote d'Ivoire 2007 | 0.44 (0.28, 0.70)** | 1.16 (0.82, 1.63) | 0.74 (0.40, 1.39) | 1.27 (0.80, 2.01) | 1.02 (0.46, 2.24) | 1.30 (0.70, 2.41) |
| Cameroon 2009 | 1.36 (0.93, 1.97) | 1.63 (1.18, 2.26)** | 1.11 (0.63, 1.96) | 1.01 (0.67, 1.52) | 0.84 (0.48, 1.46) | 1.56 (0.97, 2.50) |
| Kenya 2007 | 1.55 (1.07, 2.23)* | 0.98 (0.68, 1.41) | -- | 1.04 (0.78, 1.40) | 1.01 (0.69, 1.49) | 1.62 (1.01, 2.60)* |
| Kenya 2010 | 1.37 (0.97, 1.94) | 1.10 (0.75,1.62) | -- | 0.64 (0.41, 1.00) | 0.98 (0.61, 1.55) | 1.55 (1.01, 2.39)* |
| Liberia 2011 | 2.91 (2.04, 4.14)** | 1.29 (0.86, 1.95) | 0.55 (0.34, 0.88)* | 1.12 (0.70, 1.79) | 1.83 (1.05, 3.19)* | -- |
| Malawi 2016 | 1.14 (0.74, 1.75) | 1.91 (1.31, 2.79)** | 1.49 (0.31, 7.23) | 1.12 (0.64, 1.96) | 2.57 (1.05, 6.27)* | 0.99 (0.59, 1.67) |
| Colombia 2010 | 0.69 (0.49, 0.96)* | 1.22 (0.93, 1.59) | 0.91 (0.62, 1.32) | 1.09 (0.74, 1.60) | 1.35 (0.71, 2.56) | 1.02 (0.77, 1.35) |
| Ecuador 2012 | 0.76 (0.52, 1.10) | 1.14 (0.90, 1.45) | 0.98 (0.72, 1.33) | 1.14 (0.73, 1.77) | 1.06 (0.56, 2.00) | 1.13 (0.79, 1.62) |
| Mexico 2006 | 1.04 (0.67, 1.62) | 1.32 (0.95, 1.83) | 1.09 (0.79, 1.51) | 0.75 (0.51, 1.09) | 1.00 (0.43, 2.32) | 1.15 (0.84, 1.57) |
| Mexico 2012 | 0.61 (0.45, 0.83)** | 1.46 (1.12, 1.92)** | 1.13 (0.90, 1.43) | 1.17 (0.91, 1.50) | 1.03 (0.69, 1.54) | -- |
| Nicaragua 2005 | 0.81 (0.61, 1.08) | 1.68 (1.20, 2.35)** | 0.56 (0.29, 1.09) | -- | -- | 1.02 (0.68, 1.53) |
| USA 2006 | 0.76 (0.51, 1.13) | 1.06 (0.71, 1.59) | -- | 0.78 (0.53, 1.15) | 0.77 (0.44, 1.36) | 0.66 (0.43, 1.02) |
| Afghanistan 2013 | 1.48 (0.84, 2.62) | 0.84 (0.46, 1.51) | -- | 0.69 (0.35, 1.38) | 0.63 (0.31, 1.30) | -- |
| Azerbaijan 2013 | 0.65 (0.44, 0.98)* | 1.53 (1.10, 2.13)* | 0.91 (0.63, 1.33) | 1.20 (0.80, 1.81) | 0.90 (0.53, 1.55) | -- |
| Georgia 2009 | 0.56 (0.45, 0.71)** | 1.13 (0.92, 1.39) | 0.97 (0.74, 1.28) | -- | -- | -- |
| Mongolia 2006 | 0.63 (0.31, 1.29) | 0.85 (0.43, 1.67) | 0.80 (0.41, 1.58) | -- | -- | 1.36 (0.57, 3.25) |
| Pakistan 2011 | 0.76 (0.65, 0.89)** | 1.00 (0.86, 1.16) | 0.93 (0.76, 1.13) | 1.04 (0.85, 1.26) | 1.31 (1.01, 1.71)* | 1.14 (0.92, 1.42) |

^1^Values represent odds ratio (95% confidence interval); each value is adjusted for all other characteristics shown. Surveys are listed in alphabetical order within geographic categories, consistent with Figure 2. Surveys for which the number of cases of overweight or obesity was < 10 were excluded (Cambodia and Laos). Missing cells indicate that the variable was unavailable in the dataset, or that one level of the variable had 0 observations. * indicates P < 0.05; ** indicates P < 0.01

^2^Educational level of the head of household used because maternal education was not available in Colombia, Mexico 2006, and the United States

^3^SES was recoded for the Philippines to be a 2-level categorical variable combining middle and high SES categories. SES, socioeconomic status.
